# Lipin 1 modulates mRNA splicing during fasting adaptation in liver

**DOI:** 10.1172/jci.insight.150114

**Published:** 2021-09-08

**Authors:** Huan Wang, Tracey W. Chan, Ajay A. Vashisht, Brian G. Drew, Anna C. Calkin, Thurl E. Harris, James A. Wohlschlegel, Xinshu Xiao, Karen Reue

**Affiliations:** 1 Human Genetics, David Geffen School of Medicine,; 2 Bioinformatics Interdepartmental Program and; 3 Biological Chemistry, University of California, Los Angeles, California, USA.; 4 Baker Heart and Diabetes Institute, Melbourne, Victoria, Australia.; 5 Central Clinical School, Monash University, Melbourne, Victoria, Australia.; 6 Baker Department of Cardiometabolic Health, University of Melbourne, Parkville, Victoria, Australia.; 7 Pharmacology, University of Virginia, Charlottesville, Virginia, USA.; 8 Molecular Biology Institute and; 9 Integrative Biology and Physiology, University of California, Los Angeles, California, USA.

**Keywords:** Metabolism, Genetic diseases, Molecular biology, Mouse models

## Abstract

Lipin 1 regulates cellular lipid homeostasis through roles in glycerolipid synthesis (through phosphatidic acid phosphatase activity) and transcriptional coactivation. Lipin 1–deficient individuals exhibit episodic disease symptoms that are triggered by metabolic stress, such as stress caused by prolonged fasting. We sought to identify critical lipin 1 activities during fasting. We determined that lipin 1 deficiency induces widespread alternative mRNA splicing in liver during fasting, much of which is normalized by refeeding. The role of lipin 1 in mRNA splicing was largely independent of its enzymatic function. We identified interactions between lipin 1 and spliceosome proteins, as well as a requirement for lipin 1 to maintain homeostatic levels of spliceosome small nuclear RNAs and specific RNA splicing factors. In fasted *Lpin1*^–/–^ liver, we identified a correspondence between alternative splicing of phospholipid biosynthetic enzymes and dysregulated phospholipid levels; splicing patterns and phospholipid levels were partly normalized by feeding. Thus, lipin 1 influences hepatic lipid metabolism through mRNA splicing, as well as through enzymatic and transcriptional activities, and fasting exacerbates the deleterious effects of lipin 1 deficiency on metabolic homeostasis.

## Graphical abstract

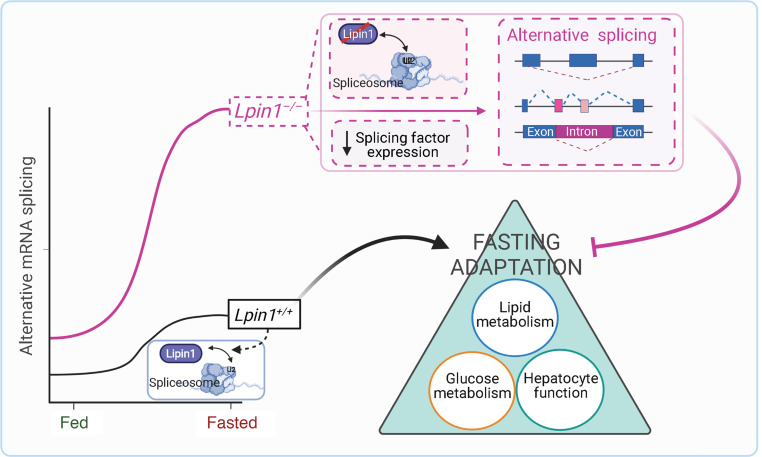

## Introduction

The regulation of lipid storage in mammalian tissues is critical for metabolic homeostasis. Excessive or inadequate triglyceride storage is associated with insulin resistance, fatty liver disease, and dyslipidemia (1). The lipin proteins (lipin 1, lipin 2, and lipin 3) perform a key reaction in the synthesis of triglycerides and phospholipids through their phosphatidate phosphatase (PAP) activity, which converts phosphatidic acid to diacylglycerol at the endoplasmic reticulum (ER) membrane (2, 3).

In addition to lipin 1 PAP activity, lipin 1 transits to the nucleus, where it influences the activity of several metabolic transcription factors. These include key regulators of fatty acid oxidation during fasting, such as PPARα and PPARγ coactivator 1α (PGC-1α; ref. 4). Lipin 1 transcriptional coactivator activity appears not to require PAP function, but it does require a hydrophobic motif (LXXIL) located downstream of the PAP active site to mediate protein-protein interactions between lipin 1 and transcription factors (4). Lipin 1 in the nucleus also leads to reduced levels of a key lipogenic transcription factor, sterol regulatory element binding protein 1 (SREBP1) (5). Lipin 1 nuclear translocation from the cytoplasm is regulated by several factors, including interaction with 14-3-3 proteins and sumoylation (6, 7). Thus, lipin proteins modulate cellular lipid homeostasis through the enzymatic conversion of lipid intermediates, as well as through interactions with transcription factors that regulate lipogenic and fatty acid oxidation gene expression.

Lipin proteins are required for human health. Lipin 1 deficiency causes recurrent episodes of rhabdomyolysis and myoglobinuria in children, with about a 10% mortality rate, likely promoted by acute kidney failure, cardiac arrhythmia, and hyperkalemia (8–11). Rhabdomyolytic bouts can be triggered in adults with lipin 1 deficiency, as well (12–14). Disease episodes in lipin 1–deficient individuals are triggered by metabolic stressors such as fasting, extreme exercise, or fever (12–15). Notably, prolonged fasting is also a trigger for other genetic deficiencies that cause rhabdomyolytic disease, such as deficiencies in fatty acid oxidation enzymes (16). There is no treatment for lipin 1 deficiency. Current interventions during acute episodes aim to alleviate symptoms through aggressive fluid and electrolyte replacement, shortened fasting periods, dietary regimens that restrict fat and/or increase carbohydrate intake, and monitoring for hyperkalemia and cardiac arrhythmias (12, 13, 15, 17). Current recommendations for long-term management of lipin 1 deficiency are to reduce fasting periods (including drinking high carbohydrate supplement drinks when meals are not possible), avoid excessive exercise, and prevent fever (12, 13, 15, 17).

Based on the role of fasting as a trigger for disease episodes in lipin 1 deficiency, we hypothesized that lipin 1 has a critical role in metabolic adaptation to fasting. Consistent with this, studies in lipin 1–deficient mice have demonstrated that lipin 1 is required for normal metabolic fuel switching between fasting and feeding (18). Stable-isotope flux analysis revealed impaired hepatic glucose production in the fasted state and increased glycogen storage and fatty acid synthesis during the fed state, likely to compensate for glucose production during the fasted state. These alterations in glucose and lipid metabolism in *Lpin1*^–/–^ mice are achieved, in part, by altered gene expression of hepatic gluconeogenic and fatty acid oxidation genes in *Lpin1*^–/–^ compared with WT mice (18).

We sought to identify critical activities of lipin 1 function in the fasted compared with the fed state. We determined that lipin 1 is required for the maintenance of mRNA splicing fidelity during fasting adaptation. This requires the nonenzymatic function of lipin 1 and is mediated by lipin 1 interaction with spliceosome components and regulation of RNA binding proteins.

## Results

### Fasting alters mRNA levels and induces widespread alternative splicing in Lpin1^–/–^ liver.

**Figure 1:**
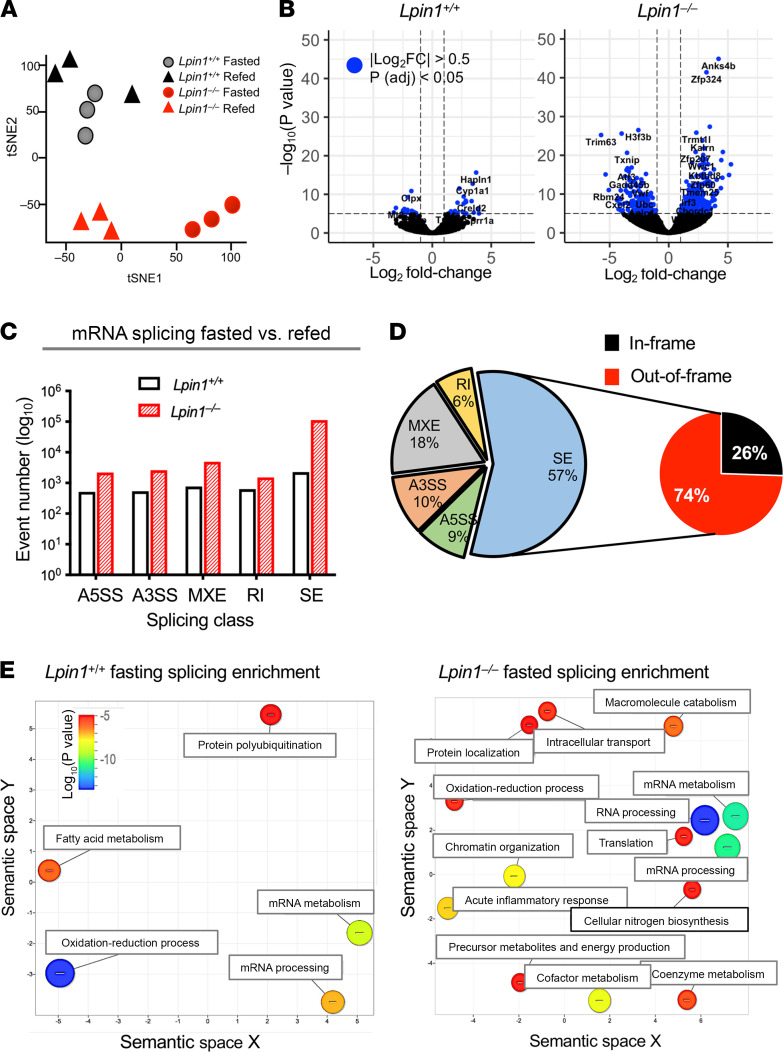
Fasting alters mRNA levels and splicing patterns in lipin 1–deficient liver. (**A**) t-SNE visualization of top 1000 differentially expressed genes with highest variance in liver of fasted and refed *Lpin1^+/+^* or *Lpin1^–/–^* mice (*n =* 3). Each symbol represents an individual mouse. (**B**) Volcano plots of differentially expressed genes in liver of fasted compared with refed *Lpin1^+/+^* or *Lpin1^–/–^* mice. Blue dots represent differential expression with an absolute value of the Log_2_ fold-change > 0.05 and adjusted *P <* 0.05 (Benjamini-Hochberg corrected). Differentially expressed genes are listed in Supplemental Table 1. (**C**) The number of alternatively spliced transcripts in fasted compared with refed *Lpin1^+/+^* or fasted compared with refed *Lpin1^–/–^* liver. Five splice patterns were assessed: A5SS, alternative 5′ splice site; A3SS, alternative 3′ splice site; MXE, mutually exclusive exons; RI, retained intron; and SE, skipped exon. (**D**) Classes of aberrant splicing events in fasted *Lpin1*^–/–^ compared with fasted *Lpin1*^+/+^ liver. Of the SE events, 74% are predicted to result in frame shifts in protein coding sequence in *Lpin1*^–/–^ liver. (**E**) Enrichment analysis (via GO term) of genes undergoing alternative skipped exon events in liver of fasted compared with refed *Lpin1^+/+^* or *Lpin1^–/–^* mice. Bubble color indicates log_10_ (*P* value); bubble size indicates the frequency of GO term in the Gene Ontology database.

To understand the role of lipin 1 in maintenance of metabolic homeostasis during the fasted state, we performed high-coverage RNA sequencing (RNA-Seq) in liver from *Lpin1^–/–^* and isogenic WT (*Lpin1*^+/+^) mice under fasting conditions (16 hours) or fed conditions (refeeding for 5 hours following a 16-hour fast). Visualization of RNA-Seq data sets by t-distributed stochastic neighbor embedding (t-SNE) plot showed separation based on *Lpin1* genotype, as well as separation between fasted and refed *Lpin1*^–/–^ liver (Figure 1A). Analysis of individual gene expression values revealed that fasting altered the expression of nearly 10-fold more genes in *Lpin1*^–/–^ liver compared with *Lpin1*^+/+^ liver (Figure 1B and Supplemental Table 1; supplemental material available online with this article; https://doi.org/10.1172/jci.insight.150114DS1). Compared with the fed state, fasting in *Lpin1*^+/+^ liver led to altered expression of genes involved in cholesterol and amino acid metabolism, whereas in *Lpin1*^–/–^ liver, fasting affected expression of mRNA processing, circadian entrainment, and various signaling pathway genes (Supplemental Figure 1A). A comparison of the 2 genotypes during fasting showed decreased fatty acid oxidation gene expression in *Lpin1*^–/–^ compared with *Lpin1*^+/+^ liver, whereas during refeeding, *Lpin1*^–/–^ differed from *Lpin1*^+/+^ in cholesterol biosynthesis and urea cycle gene expression (Supplemental Figure 1B).

Fasting is known to influence alternative mRNA splicing in liver, which may have a role in adaptation to the fasting/feeding transition (19–21). We assessed whether fasting impacts mRNA alternative splicing differentially in *Lpin1*^–/–^ compared with *Lpin1*^+/+^ liver. We evaluated 5 mRNA splicing patterns (alternative 5′ or 3′ splice sites, mutually exclusive exons (MXEs), retained introns (RIs), and skipped exons (SEs); Supplemental Figure 2). Consistent with previous reports (21), fasting led to alternative splicing events in liver of WT (*Lpin1*^+/+^ mice) (Figure 1C). Notably, *Lpin1*^–/–^ liver exhibited higher levels of alternative splicing than *Lpin1*^+/+^ liver in response to fasting (Figure 1C). For example, in the SE category, *Lpin1*^–/–^ liver had 1 × 10^5^ events compared with 1 × 10^3^ events in *Lpin1*^+/+^ liver in the fasted compared with fed states (Figure 1C and Supplemental Table 2). Of the differential exon skipping events induced in fasting compared with fed *Lpin1*^–/–^ liver, the majority are expected to generate out-of-frame transcripts that would alter the amino acid sequence or introduce a premature stop codon in the corresponding protein product (Figure 1D). In both WT and lipin 1–deficient liver, transcripts with fasting-induced alternative splicing were enriched in metabolic processes such as fatty acid metabolism, oxidation-reduction reactions, and gene expression processes; more categories showed enrichment in lipin 1–deficient liver due to the greater number of transcripts affected (Figure 1E).

**Figure 2:**
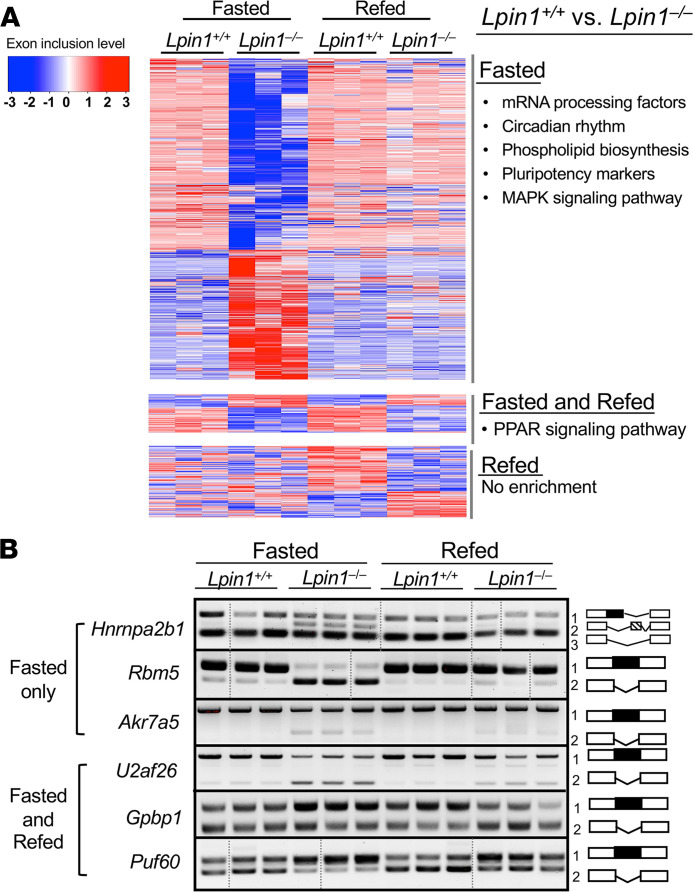
Splicing abnormalities in lipin 1–deficient liver differ in fasted and refed conditions. (**A**) Heatmap of the alternative skipped exon (SE) events in *Lpin1^+/+^* and *Lpin1^–/–^* liver in fasted and refed conditions. The scale at left shows degree of exon inclusion as the SD from the mean inclusion levels, with red indicating increased exon inclusion and blue indicating reduced exon inclusion. Heatmap is divided into 3 classes of altered splicing events in *Lpin1*^–/–^ liver: those occurring exclusively in fasted conditions, those in both fasted and refed conditions, and those exclusively in refed conditions. Functional enrichment categories of mRNAs with altered splicing in *Lpin1*^–/–^ liver under each of the conditions is indicated at right. (**B**) Splicing patterns for selected genes from heatmap in **A** visualized by RT-PCR using PCR primers that span alternatively included exons (*n =* 3). Splicing patterns that give rise to each band on agarose gels are shown at right. All samples shown in a row were run on the same gel; samples that were on the same gel but not in adjacent lanes are indicated by vertical lines. Full, uncut gels are provided in online supplemental material.

To better characterize the impact of alternative mRNA splicing in fasted *Lpin1*^–/–^ liver, we focused on SE events because they are the most abundant and the majority (74%) are predicted to lead to altered protein products. These were enriched for transcripts encoding RNA processing proteins (adjusted *P* < 1 × 10^–7^), circadian rhythm regulation (adjusted *P <* 0.02), phospholipid biosynthesis (adjusted *P <* 0.05), cell pluripotency/differentiation (adjusted *P <* 0.03), and mitogen-activated protein (MAP) kinase signaling (adjusted *P <* 0.03; Figure 2A and Supplemental Table 3). Remarkably, refeeding *Lpin1^–/–^* mice after fasting led to normalization of approximately 75% of transcripts with alternative exon splicing (Figure 2A). However, a small set of transcripts had alternative SEs in *Lpin1*^–/–^ compared with *Lpin1*^+/+^ liver in both the fasted and refed states, and these were enriched for function in the PPAR signaling pathway (adjusted *P <* 0.05). A small number of transcripts exhibited alternative exon inclusion in *Lpin1*^–/–^ liver exclusively in the refed state; these showed no functional enrichment (Figure 2A).

We verified splicing patterns detected by RNA-Seq for representative transcripts using conventional reverse transcription PCR (RT-PCR) with primers that flank differentially spliced exons (Figure 2B). The splicing patterns recapitulated the RNA-Seq results. Figure 2B shows examples of mRNAs with aberrant splicing in *Lpin1*^–/–^ liver exclusively under fasted conditions (e.g., the RNA binding proteins *Hnrnpa2b1* and *Rbm5* and the aldoketoreductase *Akr7a5*), or in both fasted and refed states (e.g., the RNA binding factors *U2af26* and *Puf60* and promoter binding factor *Gpbp1*). These data also illustrate that the proportion of a transcript for a particular gene that shows alternative splicing in fasted *Lpin1*^–/–^ liver varies from about 10%–20% of the total (e.g., *Hnrnpa2b1*) to more than 50% (e.g., *Rbm5*).

### Lipin 1 PAP-independent activity influences alternative mRNA splicing in hepatocytes.

The fasting-induced alterations in lipin 1–deficient liver could result from a direct requirement for lipin 1 to maintain mRNA splicing fidelity, or from a secondary effect that lipin 1 deficiency elicits in liver. We sought to determine if lipin 1 plays a direct role, such that acute reduction in lipin 1 levels impacts splicing. Furthermore, we sought to establish whether the PAP enzymatic function of lipin 1 is required for its effect on mRNA splicing.

**Figure 3:**
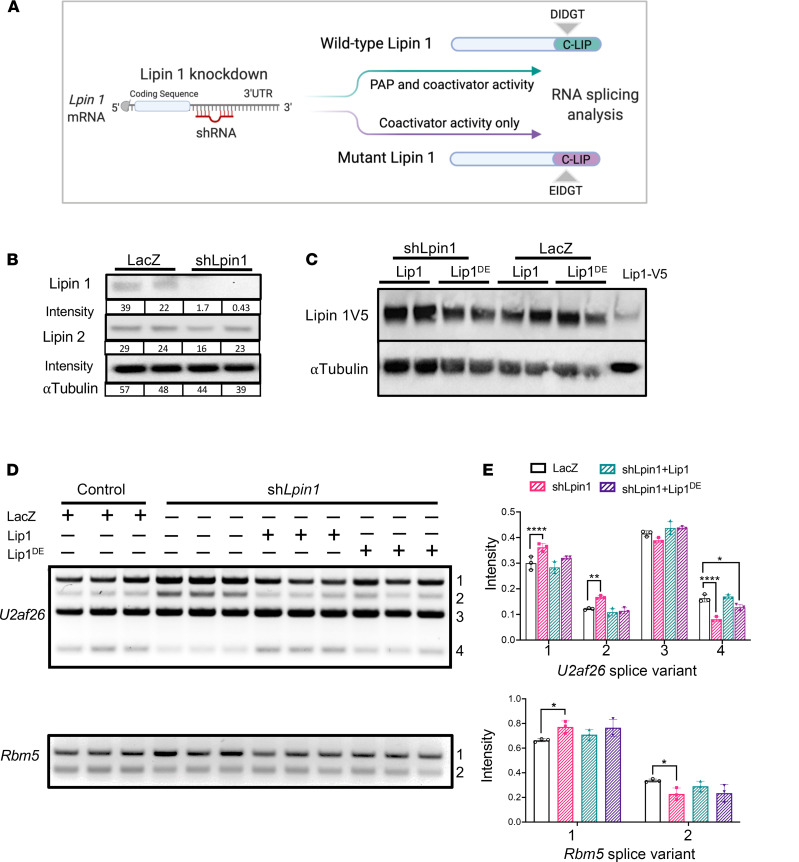
Lipin1 PAP-independent activity modulates mRNA splicing. (**A**) Experimental design to assess effect of acute lipin 1 inhibition on mRNA splicing fidelity, and the requirement for lipin 1 PAP or coactivator function. Cells treated with short hairpin RNA (shRNA) directed against lipin 1 mRNA 3′-UTR were subsequently complemented with either WT lipin 1 (PAP and coactivator activity) or mutant lipin 1 (coactivator activity only). RNA splicing pattern was assessed by RT-PCR. (**B**) Immunoblot shows that adenoviral vector expressing sh*Lpin1* reduces lipin1 protein levels but has negligible effect on lipin 2 protein levels in Hepa1-6 cells. LacZ, adenovirus vector expressing lacZ as a negative control. (**C**) Comparable protein expression levels of WT lipin 1 (Lip1) and lipin 1^D679E^ mutant protein (Lip1^DE^) via cDNA transfection after treatment of cells with sh*Lpin1*. (**D** and **E**) RNA splicing following acute lipin 1 knockdown and knockdown followed by complementation with Lip1 or Lip1^DE^. *U2af26* and *Rbm5* splicing was assessed by RT-PCR (**D**), and splice variants quantitated by densitometry (**E**) (*n =* 3). Bars in **E** represent mean ± SD; bars with different letters are significantly different from one another at *P <* 0.05 via Tukey’s HSD post hoc comparison.

We knocked down lipin 1 levels in a hepatic cell line and then complemented cells with plasmids expressing either WT lipin 1 (with both phosphatase and coactivator function) or with mutant lipin 1 that retains only coactivator function (*Lpin1*^D679E^, which inactivates the phosphatase active site; refs. 4, 22) (Figure 3A). For these studies, we used shRNA directed against the lipin 1 mRNA 3′ untranslated region to target endogenous transcripts and complemented cells with lipin 1 expression vectors that lack the shRNA target sequence. Treatment with sh*Lpin* reduced lipin 1 protein levels > 20-fold without substantially altering lipin 2 protein levels (Figure 3B). Expression of WT lipin 1 and lipin 1^D679E^ complementation vectors achieved similar levels of lipin 1 protein (Figure 3C). Adenovirally mediated expression of sh*Lpin* caused alternative splicing compared with cells infected with a control viral vector expressing LacZ, as illustrated for *U2af26* and *Rbm5* (Figure 3, D and E; pink bars compared with white bars). Following knockdown with sh*Lpin*, complementation with WT *Lpin1* expression plasmid restored splice patterns to those indistinguishable from the control (Figure 3, D and E; green bars compared with white bars). Complementation with *Lpin1*^D679E^, which lacks PAP activity, normalized the levels of most splice forms (Figure 3, D and E; purple bars compared with white bars). These results indicate that PAP-independent action of lipin 1 influences alternative mRNA splicing.

To rule out potential off-target effects of the sh*Lpin* that was used in the experiments described above, we performed lipin 1 knockdown using an antisense oligonucleotide (ASO) that targets the *Lpin1* mRNA coding region (23). An 80% knockdown of *Lpin1* mRNA levels with the ASO led to alternative splicing, as illustrated for *U2af26* and *Hnrnpa2b1* (Supplemental Figure 3, A–D). The effects of lipin 1 knockdown on splicing were observed at all concentrations of ASO employed (Supplemental Figure 3B). We note that the splicing patterns after adenoviral delivery of shRNA were slightly different than after transfection with ASO DNA. We suspect that this is related to known effects of adenovirus infection on cellular splicing patterns (24–26). Our results demonstrate that 2 independent methods used to knock down lipin 1 expression induce alternative mRNA splicing.

### Lipin 1 interacts with components of the U2 spliceosome complex.

The PAP-independent function of lipin 1 as a transcriptional coactivator/corepressor relies upon its physical interaction with transcription factors (4, 27). We wondered whether lipin 1 also interacts with proteins that influence mRNA splicing. To assess this, we performed a screen of lipin 1–interacting proteins using proximity labeling. Lipin 1 was fused to the BirA* biotin ligase (28) and expressed in Hepa1-6 mouse hepatoma cells. The subcellular distribution of lipin 1–BirA* was consistent with that of endogenous lipin 1, being detected in cytoplasmic, membrane, and nuclear/chromatin compartments (Supplemental Figure 4). Biotin was introduced to cells expressing lipin 1–BirA* to allow labeling of interacting proteins and complexes, and biotinylated proteins were purified by affinity capture and identified by mass spectrometry. We filtered mass spectrometry data to remove endogenously biotinylated proteins (identified in controls that omitted exogenous biotin treatment), and we considered hits with a minimum of 2 unique peptides per protein and a peptide-level FDR of less than 5%.

**Figure 4:**
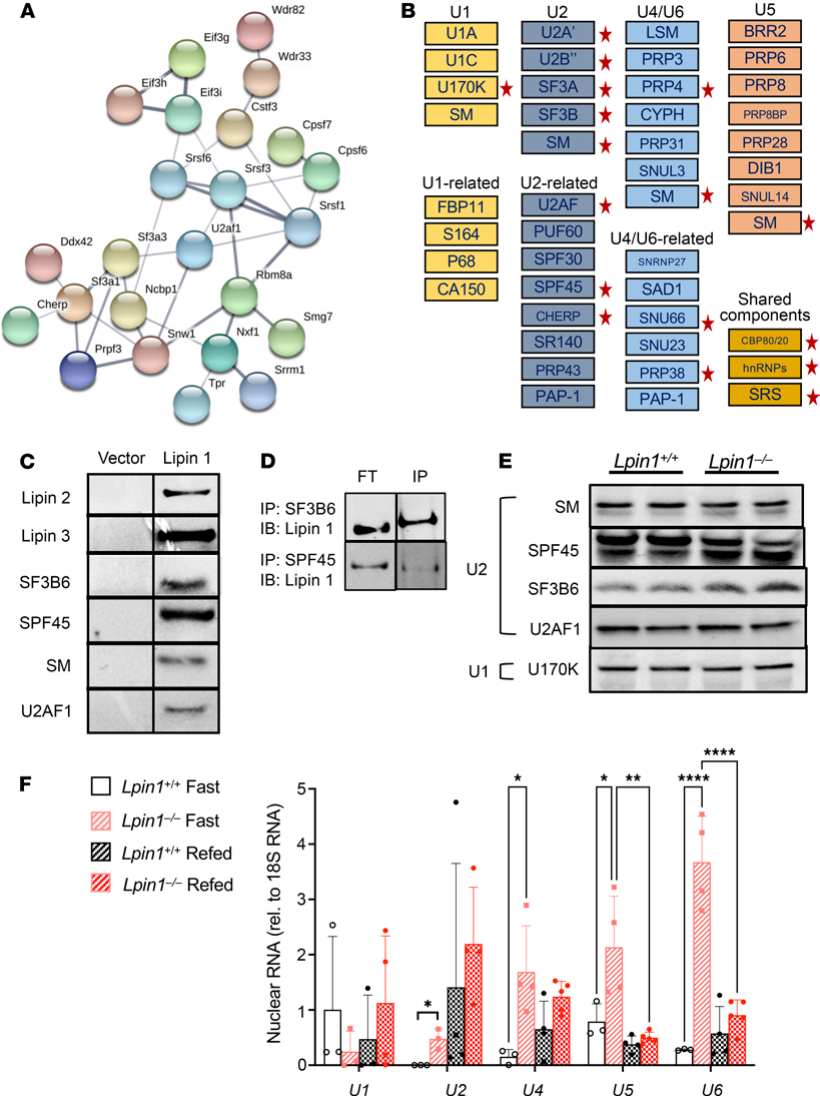
Lipin 1 interacts with mRNA processing proteins. (**A**) Network of lipin 1 interactions with mRNA processing factors (enrichment score = adjusted *P* < 1 *×* 10^–3^). Network drawn in STRING (string-db.org). (**B**) Lipin 1 associates with many proteins that are part of the U2 snRNP complex (indicated by red stars). (**C**) Validation of lipin 1 protein interactions by streptavidin pulldown followed by immunoblot. These include known lipin 1–interacting proteins (lipin 2 and lipin 3), as well as several components of the U2 snRNP. The negative control reaction was performed to demonstrate no pull-down in the presence of endogenously biotinylated proteins. Antibody information provided in Supplemental Table 8. (**D**) Coimmunoprecipitation of endogenous lipin 1 with endogenous spliceosome proteins SF3B6 and SPF45. Immunoprecipitation was performed from hepatic nuclear extracts with antibodies against spliceosome proteins and detected on blots with lipin 1 antibody. FT, flow-through; IP, immunoprecipitate; IB, immunoblot detection. (**E**) Protein levels of representative U2 and U1 spliceosome proteins in fasted hepatic nuclear extracts assessed by Western blot. (**F**) Expression levels of snRNAs U1, U2, U4, U5, and U6 in fasted and refed *Lpin1^+/+^* or *Lpin1^–/–^* liver. snRNA expression was normalized to 18S ribosomal RNA (*n =* 4). Values shown are mean ± SD; *P < 0.05 via 1-way ANOVA followed by *t* test. **P < 0.01; ****P < 0.0001.

Lipin 1 interactions included known or suspected protein partners and many components of the spliceosome. Consistent with the formation of heterodimers among the lipin family members (29, 30), lipin 1 interactions were detected with lipin 2 and lipin 3 (Supplemental Table 4). Lipin 1–interacting partners also included a network of transcription machinery proteins (Supplemental Table 4 and Supplemental Figure 4C). Pathway enrichment analysis revealed that lipin 1 associates with a large network of proteins involved in mRNA processing and the spliceosome (adjusted *P* < 1 × 10^–3^; Figure 4A). The spliceosome consists of protein and RNA components, and it assembles at splice donor and acceptor sites on nascent mRNA transcripts to catalyze the excision of intervening introns (31). Lipin 1–spliceosome associations clustered with the U2 and U2-related small nuclear ribonucleoprotein (snRNP) complex (Figure 4B; red stars). Associations were also detected with a few proteins of the U4/U6 snRNP. We verified interactions of lipin 1 with spliceosome components via streptavidin pull-down. As a positive control, streptavidin pull-down detected known interactions of lipin 1 with lipin 2 and lipin 3. Lipin 1 was also present in pull-downs with all 4 spliceosome proteins that we tested (SF3B6, SPF45, SM, and U2AF1; Figure 4C). We also confirmed interactions between endogenous lipin 1 and endogenous SF3B6 and SPF45 by coimmunoprecipitation from nuclear extracts (Figure 4D).

We hypothesized that, if lipin 1 has a role in spliceosome structure or activity, lipin 1 deficiency may lead to aberrant levels of spliceosome proteins and/or small nuclear RNAs (snRNAs) that are associated with the spliceosome complex. We assessed protein levels of several U2 spliceosome components that interact with lipin 1 in hepatic nuclear extracts and found that SPF45 and SF3B6 show slight alterations in protein levels, but other U2 components (SM, U2AF1) appear to have normal levels, as does the U1 protein, U170K (Figure 4E). Several snRNA species showed fasting-related alterations in levels in isolated chromatin (32) from *Lpin1*^–/–^ compared with *Lpin1*^+/+^ liver. Specifically, the levels of U2, U4, U5, and U6 snRNAs were altered in fasted *Lpin1*^–/–^ liver but were normalized upon refeeding (Figure 4F). Our data indicate that lipin 1 interacts with spliceosome proteins and is required for maintenance of the appropriate levels of snRNA species during fasting.

### RNA binding protein expression is dysregulated in fasted Lpin1^–/–^ liver and normalized by feeding.

**Figure 5:**
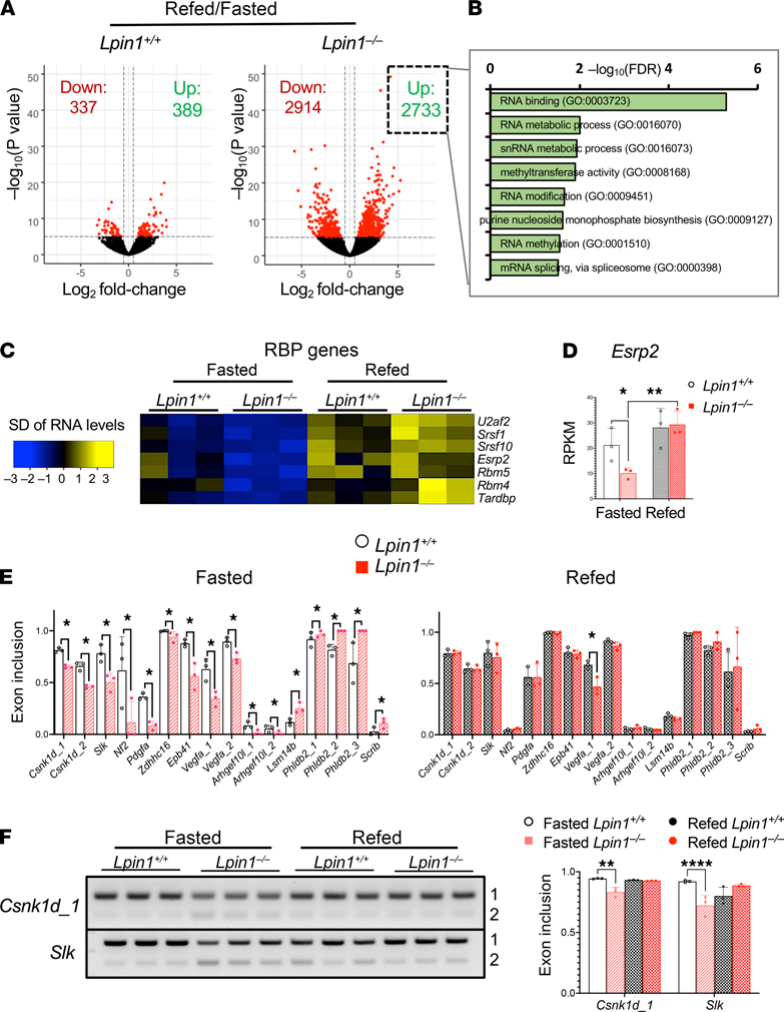
Lipin 1 deficiency leads to impaired fasting expression levels of RNA binding proteins such as ESRP2, as well as altered splicing of ESRP2 targets. (**A**) Volcano plots of differentially expressed genes in liver of refed compared with fasted *Lpin1^+/+^* or *Lpin1^–/–^* mice. Red dots represent differentially expressed genes in the refed versus fasted state (*P* < 1 *×* 10^–6^, FDR = 0.1). The numbers of differentially expressed genes that are increased (Up) or decreased (Down) by refeeding are indicated. (**B**) Genes with increased expression in refed compared with fasted *Lpin1*^–/–^ liver are functionally enriched for RNA binding and RNA processing proteins (enrichment scores indicated at top). (**C**) Heatmap of mRNA expression levels of RNA-binding protein (RBP) encoding genes are reduced in fasted *Lpin1^–/–^* liver and normalized to *Lpin1^+/+^* levels by refeeding. (**D**) Expression of the RNA binding protein ESRP2 in fasted and fed liver. Data analyzed by 2-way ANOVA followed by *t* test (*n =* 3); *P < 0.05; **P < 0.01. (**E**) ESRP2 target gene splicing in *Lpin1*^–/–^ liver is dysregulated in fasting conditions and normalized by feeding. Experimentally determined ESRP2 exon inclusion events (34, 35) were assessed in RNA-Seq data from fasted and refed *Lpin1*^–/–^ and *Lpin1*^+/+^ liver (*n =* 3). Data are shown as mean ± SD; *P < 0.05 in *Lpin1*^+/+^ versus *Lpin1*^–/–^ liver as determined by RNA-Seq analysis (FDR< 5%). (**F**) Verification of splicing of representative ESRP2 targets by standard PCR. Splice products were quantitated by densitometry (*n =* 3). Asterisks indicate significant differences analyzed by 2-way ANOVA followed by *t* test; **P < 0.01, ****P < 0.0001.

We next sought to understand what factors determine which mRNA species undergo alternative splicing in *Lpin1*^–/–^, specifically in the fasted state. We scrutinized hepatic gene expression patterns for transcripts that follow a similar pattern: they are dysregulated in *Lpin1*^–/–^ liver during the fasted state but are restored to WT levels upon feeding. We identified several mRNA transcripts that have roles in mRNA splicing and processing that exhibit this pattern (Figure 5, A and B). These include *Esrp2* (epithelial splicing regulatory protein 2), *U2af2* (U2 snRNP splicing factor U2AF 65 kDa subunit), *Srsf1* and *Srsf10* (serine and arginine-rich splicing factors), *Rbm4* and *Rbm5* (RNA binding motif proteins), and *Tardbp* (TAR DNA-binding protein 43; Figure 5C). Gene expression values for these splicing factors were reduced in *Lpin1*^–/–^ compared with *Lpin1*^+/+^ liver in the fasted state but were increased by feeding to levels similar to or higher than those in *Lpin1*^+/+^ liver (Figure 5D and Supplemental Figure 5A).

An analysis of alternatively spliced mRNAs in fasted *Lpin1*^–/–^ liver for common RNA binding motifs using RNA Map Analysis and Plotting Server 2 (rMAPS2) (33) identified ESRP2 as the top-ranked motif (Supplemental Table 5). *Lpin1*^–/–^ liver exhibited more than 7500 instances of alterative exon splicing near ESRP2 motifs in the fasted state, compared with only 1200 such instances in *Lpin1*^+/+^ liver (Supplemental Figure 5, B and C). This suggested that many of the mRNAs that are alternatively spliced in fasted *Lpin1*^–/–^ compared with *Lpin1*^+/+^ liver may be regulated by ESRP2 binding, which occurs near exon/intron boundaries and influences intron excision of transcripts that are induced during postnatal liver maturation and liver regeneration (34, 35). We assessed splicing patterns for 16 known ESRP2 targets in liver of fasted and refed mice (34, 35). Fasted *Lpin1^–/–^* mice exhibited altered exon inclusion for multiple transcripts that are involved in liver development (2 splice forms of *Csnk1d,* as well as *Slk* and *Nf2*; Figure 5E, left panel). Other classic ESRP2 targets with altered exon inclusion levels in fasted *Lpin1^–/–^* liver included *Pdgfa*, *Zdhhc16*, *Epb41*, *Vegfa*, *Arhgef10l*, *Lsm14b*, *Phldb2*, and *Scrib*. Consistent with the restoration of *Esrp2* expression upon feeding (Figure 5D), refeeding also normalized the splicing of ESRP2 targets in *Lpin1*^–/–^ liver (Figure 5, E and F). Thus, lipin 1 is required for maintenance of hepatic splicing factor gene expression and corresponding mRNA splicing patterns during the fasted state.

### Fasting promotes widespread alternative splicing of phospholipid biosynthetic genes and aberrant phospholipid levels in Lpin1^–/–^ liver.

**Figure 6:**
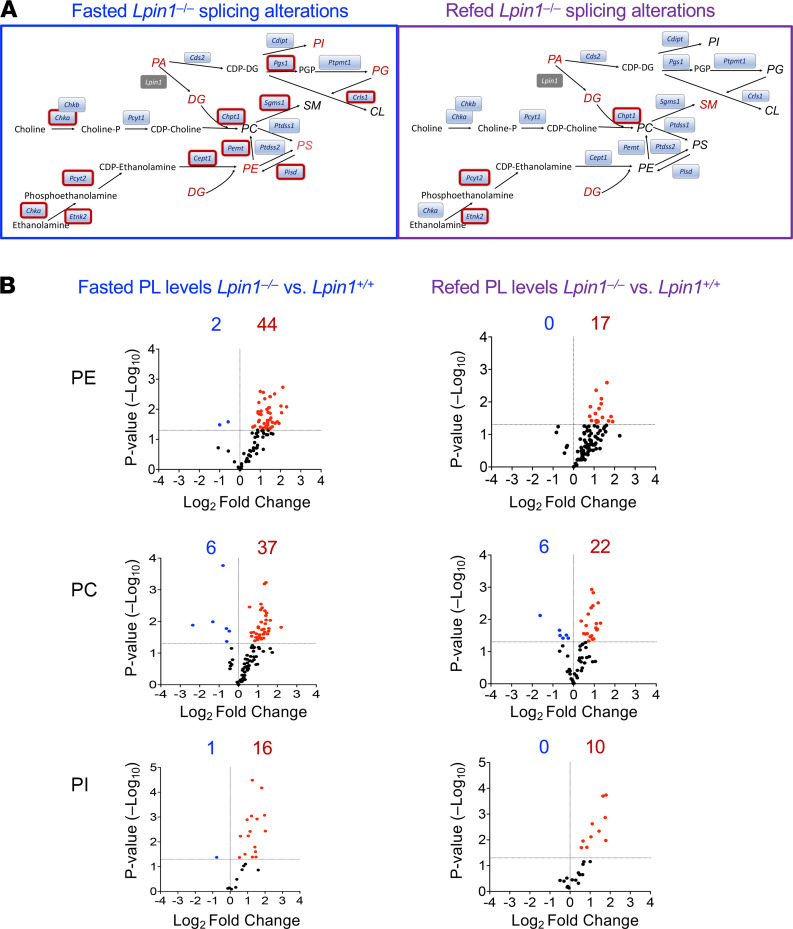
Lipin 1 deficiency promotes alternative splicing of phospholipid biosynthetic genes and dysregulated phospholipid levels in fasted liver. (**A**) Phospholipid biosynthetic enzymes and lipid products. Red outlines indicate enzymes with alternative splicing in *Lpin1*^–/–^ compared with *Lpin1*^+/+^ liver in the fasted state (left) and fed state (right). Total levels of the lipid species shown in red differ between *Lpin1*^–/–^ and *Lpin1*^+/+^ liver in the fasted (left) and fed (right) states. PA, phosphatidic acid; DG, diacylglycerol; PS phosphatidylserine; PE, phosphatidylethanolamine; PC, phosphatidylcholine; PI, phosphatidylinositol; PG, phosphoglycerate; CL, cardiolipin; SM, sphingomyelin. (**B**) Volcano plots show number of PE, PC, and PI species that differ between *Lpin1*^–/–^ and *Lpin1*^+/+^ liver in the fasted (left) and fed (right) states. Based on mean lipid levels from *n =* 5 mice/genotype.

The alternative exon splicing events in fasted *Lpin1*^–/–^ liver typically involve the utilization of an alternative exon for a proportion (10%–20%) of all transcripts from a given gene (Figure 2B and Figure 5F). This suggests that the physiological impact of a single splice variant may be modest. Even so, we hypothesized that pathways in which multiple steps undergo alternative splicing in fasted *Lpin1*^–/–^ liver may impact liver physiology. Within the pathways for synthesis of major phospholipid species, we detected alternative splicing for 10 of 17 enzymes in fasted *Lpin1*^–/–^ liver (Figure 6A, red outlines). Notably, all except 3 of the alternative splicing events in fasted *Lpin1*^–/–^ liver were normalized in the fed state (Figure 6A and Supplemental Table 6). We also assessed hepatic phospholipid levels by mass spectrometry in fasted and fed states. Numerous phosphatidylethanolamine (PE), phosphatidylcholine (PC), and phosphatidylinositol (PI) species had altered levels in fasted *Lpin1*^–/–^ compared with *Lpin1*^+/+^ liver (Figure 6B). Consistent with the known role of lipin 1 in glycerolipid synthesis, abnormalities in phospholipid levels remained in the fed state. However, fewer PE, PC, and PI species differed between *Lpin1*^–/–^ and *Lpin1*^+/+^ liver in the fed state (Figure 6B, Supplemental Figure 6, and Supplemental Table 7), in concert with normalized splicing patterns for many of the phospholipid biosynthetic enzyme transcripts in fed compared with fasted conditions.

**Figure 7:**
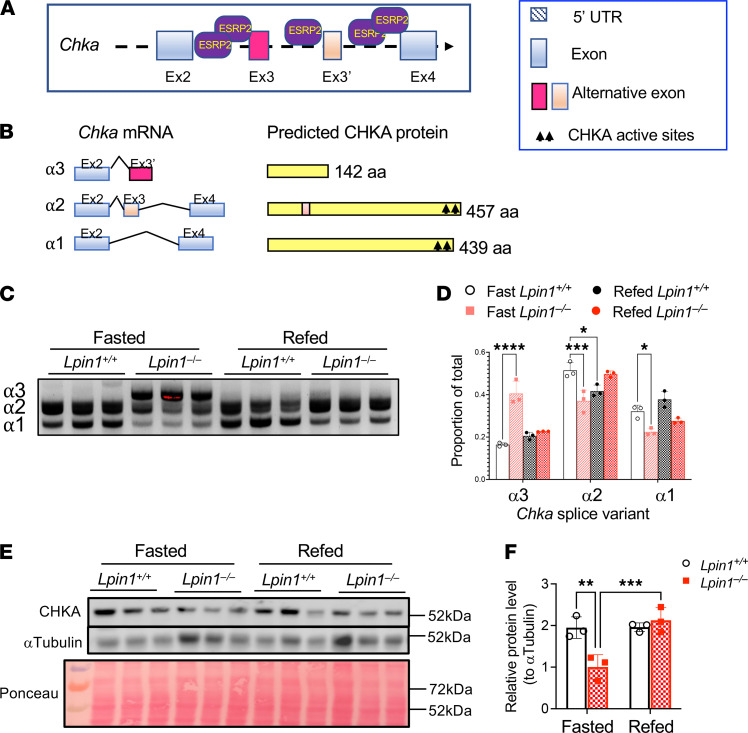
Lipin 1 deficiency promotes choline kinase α (*Chka*) alternative splicing and altered protein levels, which are more pronounced in the fasted state. (**A**) *Chka* gene region with predicted binding sites for the ESRP2 splicing factor. (**B**) *Chka* mRNA splice variants and corresponding protein products. (**C** and **D**) *Chka* splice variants in fasted and refed *Lpin1*^+/+^ and *Lpin1*^–/–^ liver assessed by RT-PCR and quantitated. Splice products were quantitated by densitometry (*n =* 3). Asterisks indicate significant differences analyzed by 2-way ANOVA followed by *t* test; *P < 0.05, ***P < 0.001, ****P < 0.0001. (**E** and **F**) CHKA protein levels by Western blot, with total protein levels shown via Ponceau staining. CHKA protein quantitation after normalization to α-tubulin. Protein levels were quantitated by densitometry and normalized by α-tubulin (*n =* 3). Asterisks indicate significant differences analyzed by 2-way ANOVA followed by *t* test; **P < 0.01, ****P < 0.0001.

It is unknown how the splice variants that occur in most of the enzymes in Figure 6A influence protein function. However, some information is available for *Chka* (encoding choline kinase α), for which 3 prominent splice variants have been previously described (36). Alternatively spliced *Chka* exons are flanked by ESRP2 binding motifs (Figure 7A; ref. 37). Alternative splicing results in enzymatically active α1 and α2 isoforms, as well as an inactive α3 isoform that results from inclusion of an alternative exon with a premature stop codon (Figure 7B). The α3 variant truncates the protein sequence and omits enzyme active sites (36). *Chka* splice variants α1 and α2 predominated in *Lpin1*^+/+^ mice in both fasted and fed states (Figure 7C). However, fasting in *Lpin1*^–/–^ mice expressed large amounts of the α3 variant, which specifies an inactive enzyme (Figure 7, C and D). Analysis of full-length CHKA protein levels verified that fasted *Lpin1*^–/–^ mice have reduced levels compared with *Lpin1*^+/+^, and these are partly normalized in the fed state (Figure 7, E and F). These findings provide a proof-of-principle illustration that alternative splice variants occurring in fasted *Lpin1*^–/–^ liver could influence protein levels and function. Although the changes in levels of any specific protein are likely to be small, the aggregate effect on proteins from widespread alternative splicing could contribute to metabolic dysregulation that occurs in lipin 1–deficient mice and humans (3).

## Discussion

**Figure 8:**
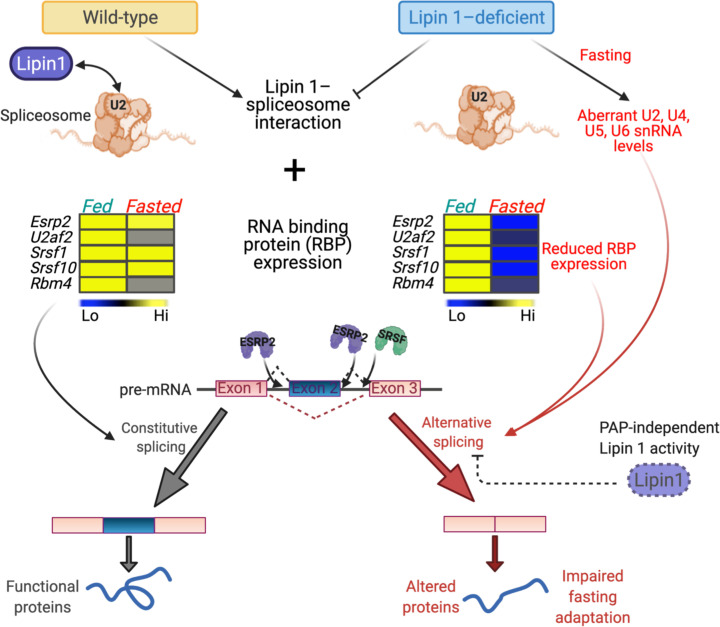
Lipin 1 influences hepatic mRNA splicing fidelity in the fasted state. Lipin 1 interacts with several components of the spliceosome, particularly with proteins of the U2 snRNP. In lipin 1–deficient liver, widespread alternative mRNA splicing occurs in the fasted state. This is associated with fasting-specific alterations in levels of spliceosome-associated snRNAs and RNA binding proteins (RBPs) with roles in mRNA splicing. Alternative splice variants in fasted *Lpin1*^–/–^ liver are enriched for RBP motifs for factors such as ESRP2 and SRSFs, which exhibit impaired expression during fasting. Alternatively spliced mRNAs are predicted to generate nonfunctional or altered function proteins in fasted *Lpin1*^–/–^ liver. The maintenance of splicing fidelity requires the PAP-independent activity of lipin 1.

The overarching function of lipin 1 is to regulate cellular and tissue lipid homeostasis. This occurs through the enzymatic role of lipin 1 in glycerolipid synthesis, as well as through its transcriptional coactivation of genes involved in fatty acid metabolism. Here, we uncover an additional mechanism by which lipin 1 influences metabolic homeostasis — by maintaining mRNA splicing fidelity in a metabolically stressful state, fasting (Figure 8).

Mechanisms for metabolic adaptation during the feeding-fasting transition include alterations in mRNA transcription and mRNA splicing, with downstream effects on protein function (19, 20). Alternative mRNA splicing is also altered in response to physiological signals, such as the circadian cycle (20), weight loss (38, 39), and metabolic pathologies such as fatty liver (40) and obesity (41). In some cases, the changes in splicing are known to be associated with altered expression levels of RNA binding proteins and splicing factors (41). We observed that WT mice adapt to fasting with alterations in both the hepatic transcriptome and mRNA splicing patterns. Our data indicate that lipin 1 normally has a role in maintaining mRNA splicing patterns, as evidenced by its interaction with splicing proteins in WT cells and the altered splicing that occurs in lipin 1–deficient liver. The exaggerated alternative splicing events that occur in fasted *Lpin1*^–/–^ liver are likely a result of loss of 1 level of regulation and may contribute to disease symptoms.

The dysregulated alternative mRNA splicing in *Lpin1*^–/–^ liver typically affects only a portion of transcripts for a given protein product and, therefore, is expected to produce modest effects on the function of any specific protein. However, the composite effect of alterations in multiple proteins that function within a particular metabolic pathway could have physiological consequences. As an illustration of this point, we detected alternative splicing in fasted *Lpin1*^–/–^ liver for the majority (10 of 17) of enzymes in the phospholipid biosynthetic pathway, and we found substantial dysregulation of numerous phospholipid species (Figure 6 and Supplemental Figure 6). In fed *Lpin1*^–/–^ liver, both mRNA splicing of these enzymes and phospholipid levels were partially normalized to more closely resemble WT. The consequence of most alternative splicing events have not been characterized for their effects on protein function. An exception is choline kinase α, for which 3 alternative splice variants have been characterized previously (36). We found that fasted *Lpin1*^–/–^ liver predominantly expressed the α3 splice variant (Figure 7C), which specifies a truncated protein without enzymatic activity (36). The *Chka* splicing pattern was normalized with feeding, and levels of full-length CHKA protein also resemble those in *Lpin1*^+/+^ liver in the refed state. Although the function of the splice variants detected for other phospholipid biosynthetic enzymes are not known, it is likely that the differential splicing in the fasted *Lpin1*^–/–^ liver may further contribute to dysregulation of phospholipid homeostasis during fasting.

The role of lipin 1 in mRNA splicing was largely independent of its PAP enzymatic function. Analogous to lipin 1 interaction with DNA-binding transcription factors, we detected lipin 1 interactions with spliceosome proteins, particularly components of the U2 complex. We also found that lipin 1 is required for the regulation of spliceosome-associated snRNA levels during fasting. Additionally, lipin 1 influences RNA binding protein gene expression, which was reduced specifically in the fasted state in lipin 1–deficient liver. Additional mechanisms may contribute to the pronounced role for lipin 1 in mRNA splicing in the fasted state. For example, lipin 1 posttranslational modifications, which are known to occur in response to insulin and other stimuli (6, 7, 42), could influence lipin 1 interactions with spliceosome components through effects on protein charge or alterations in lipin 1 subcellular localization. Further studies are warranted to investigate these possibilities.

In our analysis of all transcripts with alternative splicing in fasting *Lpin1*^–/–^ liver, motifs for the ESRP2 splicing factor were the most prevalent (Supplemental Figure 5). ESRP2 regulates a shift in the mRNA splicing program during the maturation of neonatal to adult liver in mice and humans (34, 43). Fasted *Lpin1*^–/–^ liver exhibited reduced levels of *Esrp2* expression and aberrant exon inclusion in numerous established ESRP2 target genes (Figure 5E). In the fed state, *Esrp2* expression levels and target gene splicing patterns were normalized to those present in WT mice (Figure 5D). Consistent with ESRP2 dysregulation, *Lpin1*^–/–^ mice exhibit impaired liver regeneration following partial hepatectomy (44). This has been attributed to reduced availability in *Lpin1*^–/–^ mice of adipose tissue–derived fatty acids, which are thought to be important during liver regeneration. However, our studies raise the possibility that aberrant mRNA splicing programs necessary for liver maturation may also contribute to impaired liver regeneration in lipin 1 deficiency.

Several of the splicing factors that are dysregulated in fasted *Lpin1*^–/–^ liver (Figure 5C and Supplemental Figure 5A) have been implicated in liver disease. Nonalcoholic fatty liver disease is characterized by dysregulation of SRSF1, SRSF10, and ESRP2 (45). Reduced levels of RBM4, as seen in fasted *Lpin1*^–/–^ liver, are associated with poor prognosis in hepatocellular carcinoma following hepatectomy (46). Fasted *Lpin1*^–/–^ liver also had reduced levels of TRA2B (also known as SFRS10), which was previously found to be reduced in liver of obese subjects and to regulate splicing of the human *LPIN1* gene and hepatic lipogenesis (47).

In summary, our findings reveal that lipin 1 is critical for the regulation of hepatic mRNA splicing fidelity in response to metabolic stress that occurs during fasting (Figure 8). Analogous to the role of lipin 1 in transcriptional coactivation, this role of lipin 1 appears to involve the interaction with proteins that specialize in splicing and does not require lipin 1 enzymatic function. Our findings raise the possibility that aberrant alternatively spliced mRNA transcripts and the corresponding impact on protein function contribute to the disease symptoms in lipin 1 deficiency. They also suggest that small molecules that have been useful to treat diseases characterized by widespread splicing defects (48, 49) could be potentially beneficial in lipin 1 deficiency.

## Methods

### Mice.

*Lpin1*^–/–^ (fatty liver dystrophy) mice and WT littermates (BALB/cByJ background) were from a colony maintained at UCLA that was originally established from *Lpin1*^+/–^ mice obtained from The Jackson Laboratory (001592, BALB/cByJ-*Lpin1^fld^*/J). Mice were reared in groups of 3–4/cage, at ambient temperature of 22°C–24°C with 12-hour light/12-hour dark conditions and fed a laboratory chow diet. Fasting was performed for 16 hours (1700–0900 hours) with singly caged mice at a temperature of 18°C–20°C. Refeeding was performed on fasted mice for 5 hours (0900–1400 hours) by providing chow pellets ad libitum. Male mice (aged 2–8 months) were used for RNA-Seq and lipidomic analyses; both male and female mice (aged 2–5 months) were used to validate splicing changes, effects on splicing factor protein, and snRNA levels.

### RNA-Seq and analysis.

Total RNA from liver tissue was isolated using RNeasy tissue mini kit (QIAGEN). RNA quality and quantity was determined using the TapeStation 2200 system (Agilent Technologies) before library generation. Libraries were prepared by the UCLA Neuroscience Genomics Core with Illumina standard kits (TruSeq stranded RNA v2) according to standard protocols. All samples were barcoded and sequenced with 12 samples (*n =* 3) per lane and 6 lanes in total, with HiSeq2000 4K using a 69 bp paired-end sequencing protocol to achieve approximately 150 million reads per sample. A round-robin design was implemented such that biological replicates were sequenced on different lanes, and each sample was part of more than 1 sequencing pool. The samples were demultiplexed, and forward- and reverse-read fastq files were generated for each sample.

Quality of the raw RNA-Seq reads was assessed with FastQC software, and RNA-Seq reads were aligned to mouse genome and transcriptome (GRCm38 Ensembl release 84) using HISAT2. Gene-level read counts were obtained using htseq-count. Gene expression level was quantified as reads per kilobase of transcript per million mapped reads (RPKM). Differential gene expression analysis was performed by DESeq with an FDR < 0.05 as previously described. Differential splicing events between groups of triplicate samples were identified using rMATS (50). Five major types of splicing events were assessed: SE, alternative 5′ splice site (A5SS), alternative 3′ splice site (A3SS), MXE, and RI. RNA-Seq data that include all global gene expression data and splicing data are available at Gene Expression Omnibus database (GEO; GSE160984).

### Functional enrichment analysis.

Functional pathway analysis of differentially expressed genes was performed using Enrichr (51). Functional enrichment analysis of alternative SEs events was performed using Gene Ontology enrichment analysis and visualization tool (GOrilla; ref. 52). Scatter plots were generated with REViGO (53).

### Heatmaps and hierarchical cluster analysis.

Hierarchical clustering by Euclidean distance for exon inclusion levels and differential expressed RNA-binding protein genes was performed with the Dynamic Tree Cut package using R (54). Heatmaps were generated using the heatmap.2 function from gplots (55).

### Motif enrichment analysis and RNA maps analysis.

We used the rMAPS online platform (33) to identify differential enrichment of RNA binding protein motifs in exon skipping events between fasted and refed conditions in *Lpin1^–/–^* and *Lpin1^+/+^* liver. We applied 115 known RNA binding protein motifs corresponding to characterized splicing factors (33). Motif scores were calculated for a region that included the exon body, 250 bp of upstream and downstream intron, flanking exons, and 250 bp intronic regions of flanking exons. For each motif, the *P* value for enrichment analysis was calculated based on the number of occurrences in the differentially spliced exons and the control exons through the Fisher’s exact test (right-sided) and using Benjamini-Hochberg FDR correction to adjust for multiple testing. We considered enriched motifs with an FDR < 5% and *P* < 0.01. An alternative exon was classified as a control alternative exon if it did not show any splicing change (rMATS FDR > 50%, maximum percent spliced in PSI > 15%, minimum percent spliced in PSI < 85%) and if it was from a highly expressed gene (average fragments per kilobase of transcript per million mapped reads > 5.0 in at least 1 group).

### Cell culture and transfection.

Murine Hepa1-6 hepatoma cells (American Type Culture Collection, CRL-1830) were maintained in complete DMEM with 10% FBS (Corning Inc.). All experiments were performed with cells having 2–6 passages. Cells were transfected with plasmids using BioT reagent (Bioland Scientific LLC).

### Subcellular fractionation and immunoprecipitation.

Hepa1-6 cells were fractionated into cytoplasmic, membrane-bond, soluble nuclear, and chromatin-bound nuclear fractions using the Subcellular Protein Fractionation Kit for Cultured Cells (Thermo Fisher Scientific, 78840). Studies were performed with the addition of 1 mM phenylmethylsulfonyl fluoride (PMSF) and 1× protease inhibitor cocktail (MilliporeSigma). Immunoblot analysis was performed on Hepa1-6 cell lysates or mouse liver tissue as previously described (11). Antibodies used are presented in Supplemental Table 9.

Liver nuclei were purified from freshly harvested liver. Tissue (100 mg) was minced and washed with PBS. The pellet was lysed in hypotonic buffer (10 mM Tris-HCl [pH 7.9], 1.5 mM MgCl_2_, 10 mM KCl, 1 mM dithiothreitol,1 mM PMSF, and 1× protease inhibitor cocktail) in a Dounce homogenizer (40 strokes). Nuclear pellets were collected by centrifugation (20,000*g* for 30 minutes at 4°C), washed twice in hypotonic buffer, and resuspended in nuclear extraction buffer (50 mM Tris-HCl [pH 7.9], 1 mM MgCl_2_, 1 mM DTT, 0.1% Nonidet P-40 [NP-40], 250 units/mL Benzonase [MilliporeSigma], 1 mM PMSF, and 1× protease inhibitor cocktail) with sonication. The supernatant (nuclear extract) was collected after centrifugation for 10 minutes at 4°C at 17,000*g*.

For immunoprecipitation, nuclear extracts were incubated with antibodies against spliceosome components (Supplemental Table 2) at 4°C overnight. Protein A/G-agarose beads were added for 2 hours at 4°C. Immunoprecipitates were collected by centrifugation (1500*g* for 1 minute at room temperature), washed 3 times with lysis buffer, and subjected to Western blot analysis with antibody against lipin 1.

### Lipin 1 knockdown and splicing analysis by RT-PCR.

Lipin 1 knockdown was performed with shRNA or ASO. For shRNA studies, U6 promoter-driven shRNA hairpins targeting the 3′-UTR of lipin 1 (3765-3784 of *Lpin1* transcript variant X1; accession XM_006514975) and the coding region of lipin 2 (7633-7651 of *Lpin2* transcript variant X1; accession XM_006524786) were subcloned into pAdTRACK and used to generate adenovirus (XM_006514975, https://www.ncbi.nlm.nih.gov/nuccore/XM_006514975) (XM_006524786, https://www.ncbi.nlm.nih.gov/nuccore/XM_006524786) (56). Adenovirus was packaged with poly-L–Lysine and delivered to Hepa1-6 cells for 24 hours. For complementation studies, cells were coinfected with sh*Lpin1* and adenovirus vectors expressing lipin 1 cDNAs (WT or D679E mutant lipin 1) that lack the shRNA binding site. Cells were harvested 1 day later for RNA splicing analysis by RT-PCR.

For ASO studies, cells were transfected with ASO targeting the lipin 1 coding region or a control nonspecific ASO (23) with Lipofectamine RNAiMAX (Thermo Fisher Scientific). RNA was isolated for splice variant analysis 4 days after transfection. RT-PCR was performed using PCR primers that span alternative exons (sequences in Supplemental Table 8), and products were analyzed by agarose gel electrophoresis.

### Proximity-dependent biotin identification (BioID).

Protein-protein associations were detected by fusion of lipin 1 coding sequences (57) with the BirA* biotin ligase (28) in pcDNA3.1 MCS-BirA*-HA (Addgene, 36047). Hepa1-6 cells were seeded at 200,000 cells per well in 6-well plates. The following day, cells were transfected with lipin-BirA* plasmids in BioT reagent (Bioland Scientific). Twenty-four hours after transfection, cells were supplied with fresh medium containing 100 μM Biotin (MilliporeSigma), and cell protein lysates were collected 24 hours later and supplemented with protease and phosphatase inhibitors. Prior to submitting samples for mass spectrometric analysis, successful biotinylation was confirmed by subjecting lysates to Western blot probed with streptavidin-HRP (Thermo Fisher Scientific). The level of endogenously biotinylated proteins was assessed by Western blot of lysates from untransfected cells that were treated with biotin. Antibodies used in the verification of lipin 1–interacting proteins are listed in Supplemental Table 9.

### Protein mass spectrometry.

Purified proteins bound to streptavidin beads were reduced, alkylated, and digested by sequential addition of Lys-C and trypsin proteases (58). Samples were desalted and subjected to ultra high–pressure liquid chromatography and tandem mass spectrometry. Data analysis was performed with ProLuCID and DTASelect2 implemented in the integrated proteomics pipeline IP2 (Integrated Proteomics Applications Inc.; ref. 58). Protein and peptide data were filtered using DTASelect and required a minimum of 2 unique peptides per protein and a peptide-level false-positive rate of less than 5%.

### Chromatin-associated RNA extraction and snRNA real-time RT-PCR.

To isolate chromatin-associated RNA, fresh liver tissue was homogenized and chromatin-associated RNA was isolated as described (32). Briefly, nuclei were purified and RNA isolated with TRIzol (Invitrogen). Following digestion with DNase I, RT-PCR was performed using primers complementary to mouse snRNAs (Supplemental Table 7) and normalized to 18S ribosomal RNA using SYBR Green PCR Master Mix (Bio-Rad). Absence of contaminating genomic DNA was verified by the lack of amplified products in mock reverse-transcription reactions in which no enzyme was added.

### Lipid analyses.

Lipidomic analyses were performed on hepatic lipids extracted by a modification of the Bligh and Dyer method (59). Lipid species were quantified by electrospray ionization–tandem mass spectrometry as described previously (60).

### Data availability.

The authors declare that the data supporting the findings of this study are available within the paper and its supplementary information files or are available in the public GEO database (accession GSE160984).

### Statistics.

Statistical analyses for group comparisons in Figure 5E and Supplemental Figure 3A were by *t* test. Group comparisons in Figure 4F; Figure 5D; Figure 7, D and F; Supplemental Figure 3D; and Supplemental Figure 5A were by 2-way ANOVA; significant ANOVA (*P <* 0.05) tests followed by *t* tests. All *t* tests are 2 tailed. Statistical analyses in Figure 3E were performed with Tukey’s HSD post hoc comparison. Statistics for analysis of differential RNA splicing, motif enrichment, enrichment of protein, or gene expression functional groups were as described under methodology and in figure legends. *P* values presented for RNA-Seq mRNA levels and levels of splice variants, functional enrichment, and motif enrichment analyses were all adjusted *P* values, taking into account multiple comparisons.

### Study approval.

Animal studies were performed after approval from the UCLA IACUC.

## Author contributions

HW performed mouse and cell culture experiments, interpreted omics data, and wrote the manuscript; TWC analyzed RNA-Seq data; AAV and JAW designed and performed proteomics experiments; BGD and ACC performed lipidomic analyses; TEH provided shRNA vectors; XX supervised RNA-Seq analysis; and KR designed experiments, obtained funding, and wrote the manuscript.

## Supplementary Material

Supplemental data

Supplemental tables 1-9

## Version 1 09/08/2021

Electronic publication
