# Supplementary material for: Lipin 1 modulates mRNA splicing during fasting adaptation in liver
Source: JCI Insight. 2021 Sep 8;6(17):e150114. doi: 10.1172/jci.insight.150114 (PMC8492312; doi:10.1172/jci.insight.150114)

Supplemental Figure 1

A Global hepatic gene expression: Fasted vs. Refed within genotype

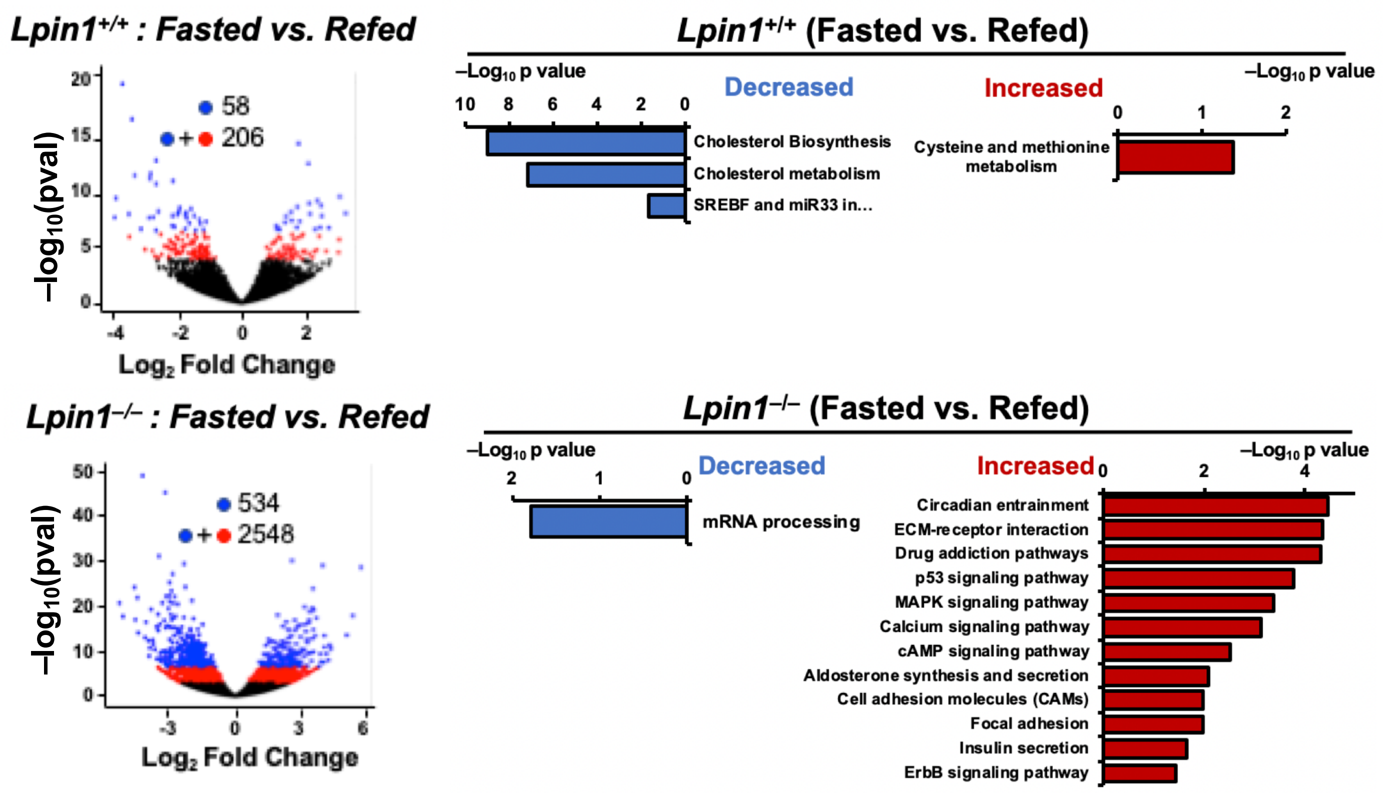

B Global hepatic gene expression: *Lpin1*–/– vs. *Lpin1*+/+ fasted or refed

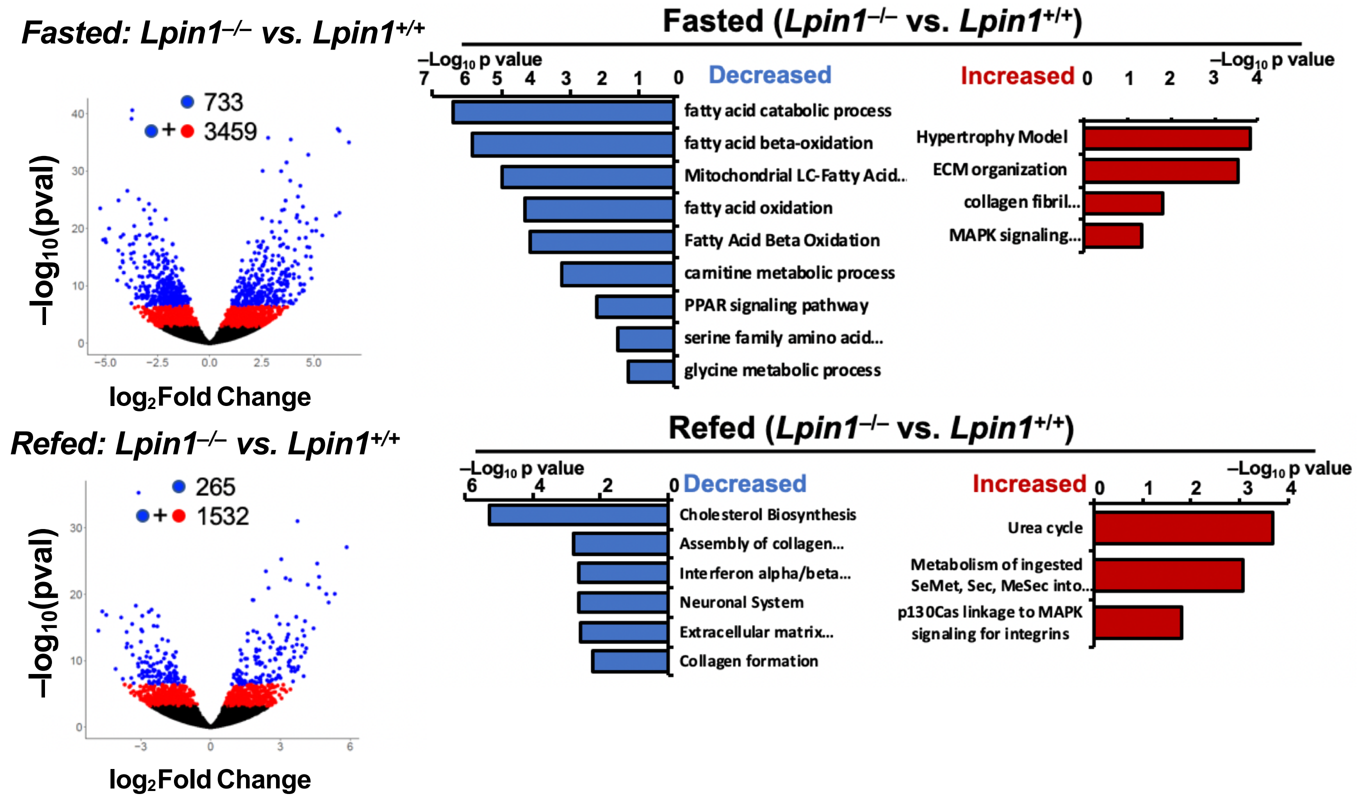

Supplemental Figure 1: Functional enrichment of differential gene expression in *Lpin1*$^{+/+}$ and *Lpin1*$^{-/-}$ liver in fasted and fed states.

A: Volcano plots of genes differentially expressed in *Lpin1*$^{+/+}$ liver in the fasted vs. refed state, or in *Lpin1*$^{-/-}$ liver in fasted vs. refed state. Mice were fasted 16 hr or fasted 16 hr and refed 5 hr. Blue dots, $p<0.01$ (Bonferroni correction); red dots, $p<0.01$ (Benjamini-Hochberg correction). Functional enrichment of differentially expressed genes was performed by Enrichr. Significance of pathway enrichment is expressed as $-\log_{10}$ (adjusted p value) as derived from comparison with random gene sets.

B. Volcano plots of genes differentially expressed in *Lpin1*$^{+/+}$ vs. *Lpin1*$^{-/-}$ liver. Gene expression and enrichment was analyzed as described in (A).

Supplemental Figure 2

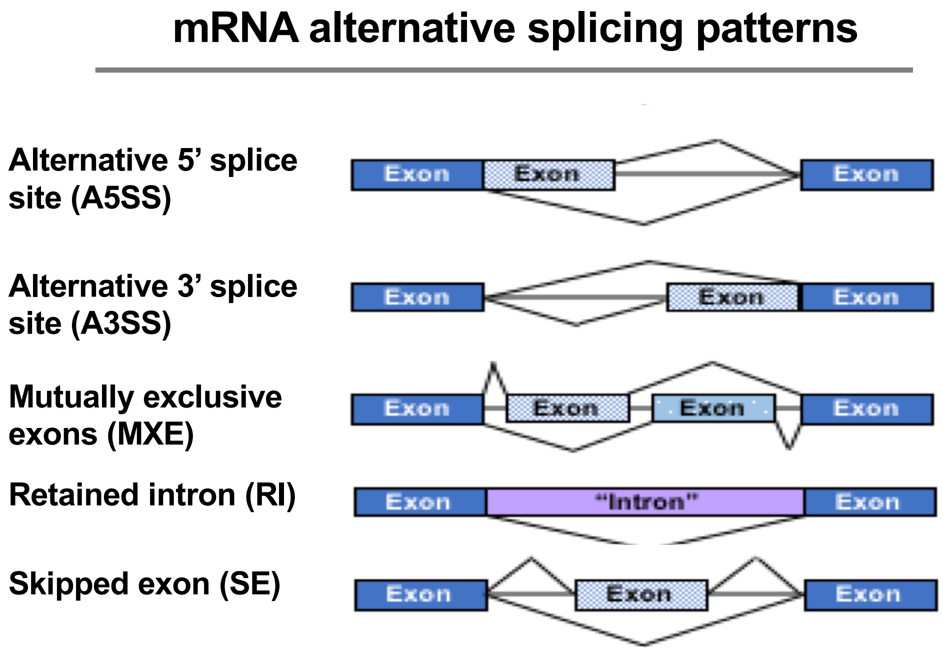

Supplemental Figure 2: Splicing classes analyzed in our study.

The 5 mRNA splice classes assessed by RNA-seq include Alternative 5' or 3' splice site (A5SS, A3SS), Mutually Exclusive Exon (MXE), Retained Intron (RI), and Skipped Exon (SE). Blue boxes indicate constitutive exons, light colored boxes are potential alternative exons, and purple box indicates an intron that is retained in mRNA.

Supplemental Figure 3

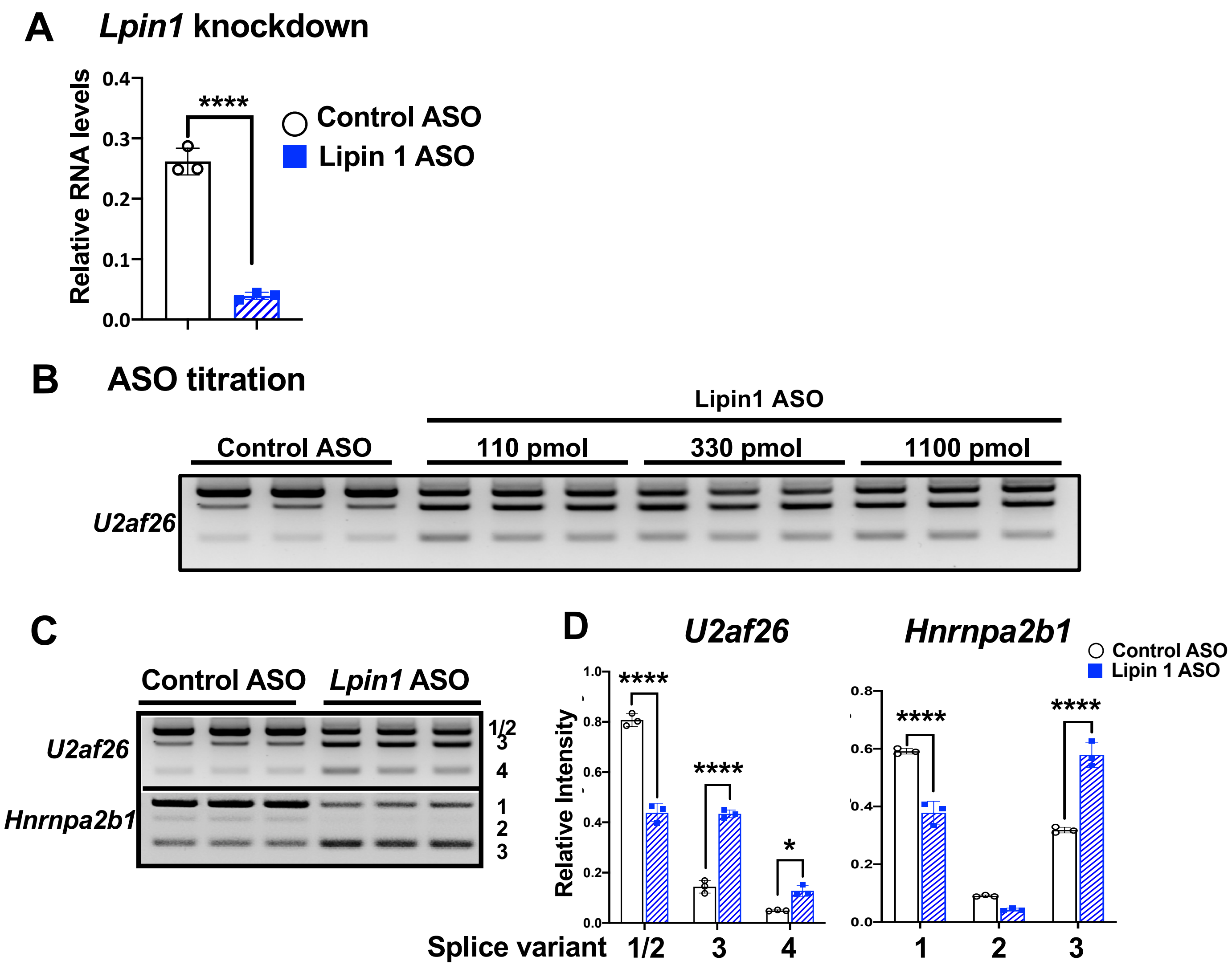

Supplemental Figure 3: Acute lipin 1 knockdown by antisense oligonucleotide alters mRNA splicing.

A: *Lpin1* mRNA levels in Hepa1-6 cells treated with antisense oligonucleotide (ASO, 110 μM) directed against the lipin 1 mRNA coding region compared to cells treated with a non-specific control ASO. mRNA expression was normalized to 18S ribosomal RNA (n=3). Bars represent mean ± SD; n=3, *, p<0.05 by t-test.

B: Titration of ASO concentration shows alterations in mRNA splicing at lowest concentration used (110 pmol) and consistent pattern across concentrations.

C–D: *Lpin1* knockdown by ASO alters mRNA splicing. Splice variants of *U2af26* and *Hnrnpa2b1* mRNAs were analyzed by RT-PCR using primers spanning alternative exons (C), and splice products were quantitated by densitometry (D). Asterisks indicate significant differences analyzed by 2-way ANOVA followed by t-test (n=3); *, p<0.05, ****, p<0.0001.

Supplemental Figure 4

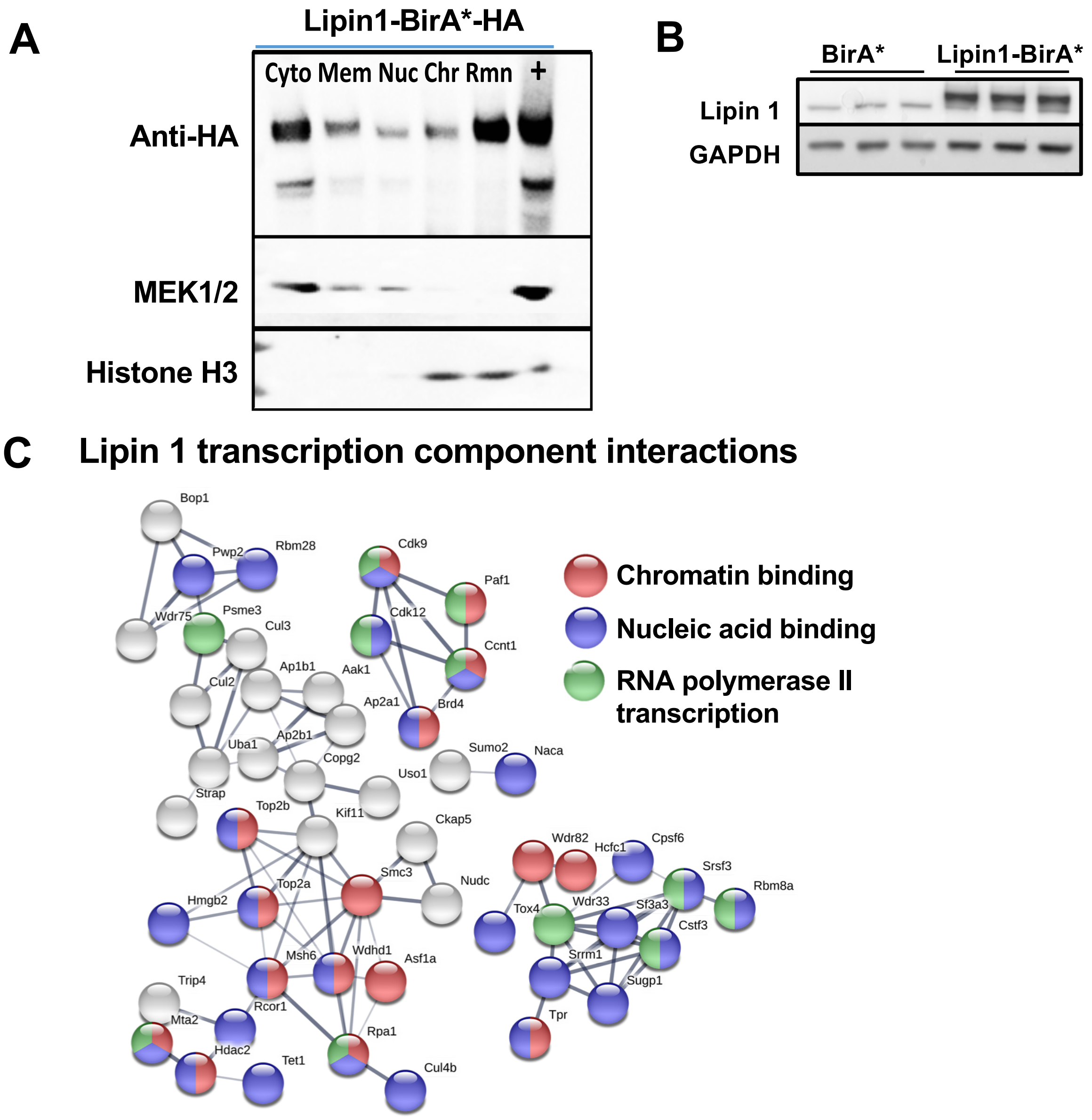

Supplemental Figure 4: Lipin 1-BirA* protein subcellular localization and lipin 1 interactions with transcription network.

A: Hepa1-6 cells transfected with lipin 1-BirA* were fractionated into five compartments: cytoplasm (Cyto), membranes (Mem), soluble nuclear fraction (Nuc), chromatin-bound nuclear fraction (Chr), and the remaining fraction (Rmn). Lipin-BirA* proteins were detected by immunoblot with anti-HA antibody. “+” represents total protein lysate. Immunoblotting of proteins with known cytoplasmic (MEK1/2) or nuclear/chromatin-localization (histone H3) was also performed.

B: Lipin 1 protein levels in Hepa1–6 cells transfected with Lipin1-BirA* vs. BirA* control vector. GAPDH, glyceraldehyde 3-phosphate dehydrogenase.

C: Network of lipin 1 interactions with transcriptional regulatory proteins, including chromatin binding proteins (red; enrichment score, adjusted $p<10^{-12}$), nucleic acid binding proteins (purple; enrichment score, adjusted $p<10^{-7}$), and RNA polymerase II transcription factors (green; enrichment score, adjusted $p<10^{-4}$). Network drawn in STRING (string-db.org).

Supplemental Figure 5

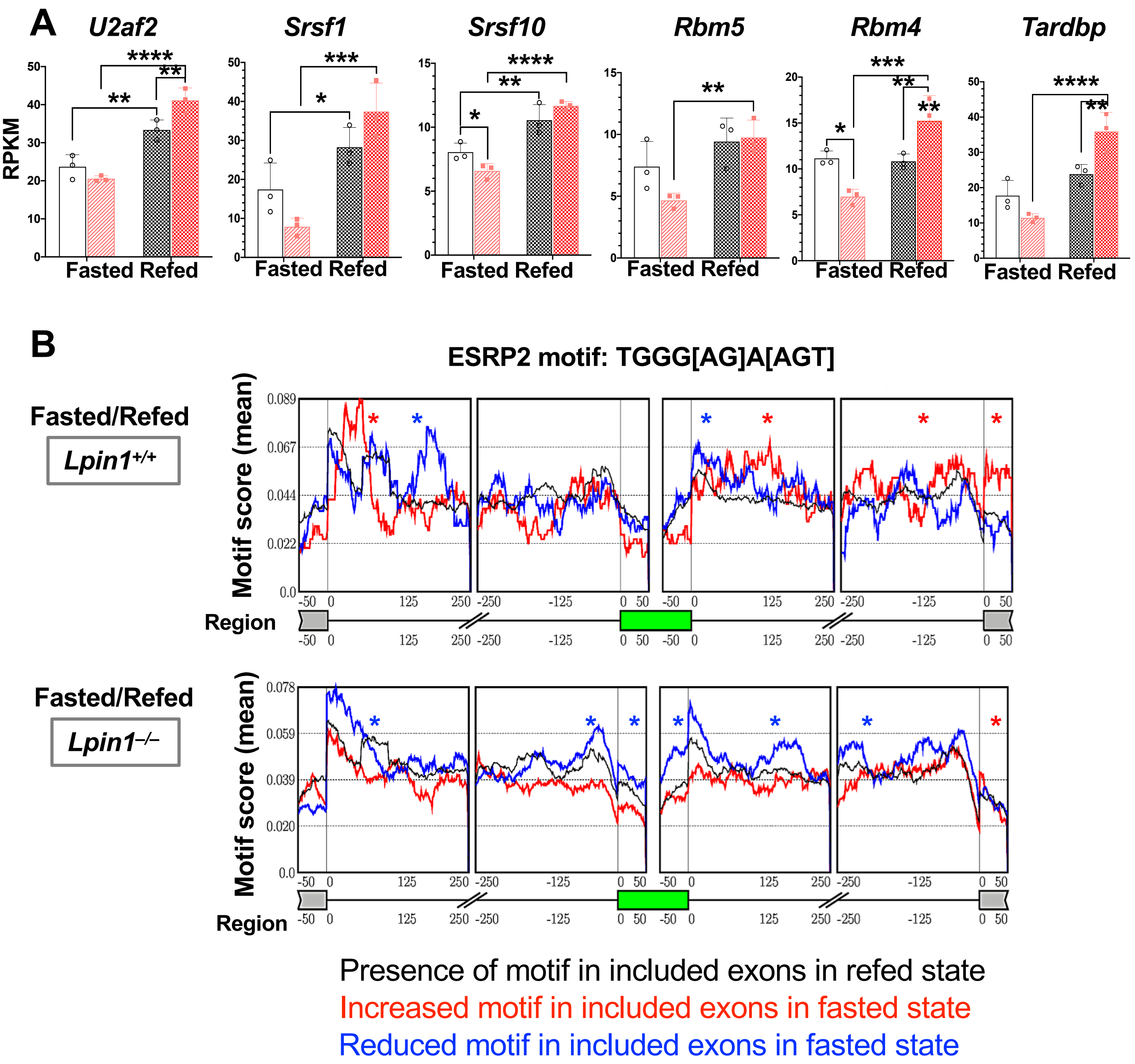

C

Number of ESRP2–enriched AS exons in fasted condition (relative to refed)	*Lpin1*$^{+/+}$	*Lpin1*$^{-/-}$
Increased ESRP2 Inclusion	500	2893
Decreased ESRP2 Inclusion	732	4777

Supplemental Figure 5: Fasting in Lipin 1-deficient liver alters RNA binding protein (RBP) gene expression and alternative exon use near ESRP2 motifs.

A: Expression levels of RBP genes shown in Fig. 5C for *Lpin1*$^{+/+}$ and *Lpin1*$^{-/-}$ mice under fasted and refed conditions. Asterisks indicate significant differences analyzed by 2-way ANOVA followed by t-test (n=3); *, p<0.05, **, p<0.01, ***, p<0.0001).

B: Differences in ESRP2 motif presence at sites of alternative exon splicing in the fasted state (red and blue lines) compared to those that do not differ in the two conditions (black line). Motif scores (y-axis) computed by rMAPS. Red asterisks (p<0.05) indicate positions relative to the alternatively spliced exon (green box) where there is an increase in ESRP2 motif occurrence in fasting compared to refed splice variants; blue asterisks indicate positions that show a decreased ESRP2 motif occurrence in fasting compared to refed splice variants. Fasted *Lpin1*$^{-/-}$ liver shows highly significant deviation from the refed state in transcripts that contain ESRP2 motifs (blue line vs. black line).

C: Number of ESRP2-enriched alternatively spliced exons in fasted compared to refed *Lpin1*$^{+/+}$ and *Lpin1*$^{-/-}$ liver. Alternatively spliced mRNAs in fasted *Lpin1*$^{-/-}$ liver are substantially enriched in ESRP2 motifs compared to *Lpin1*$^{+/+}$ liver.

Supplemental Figure 6

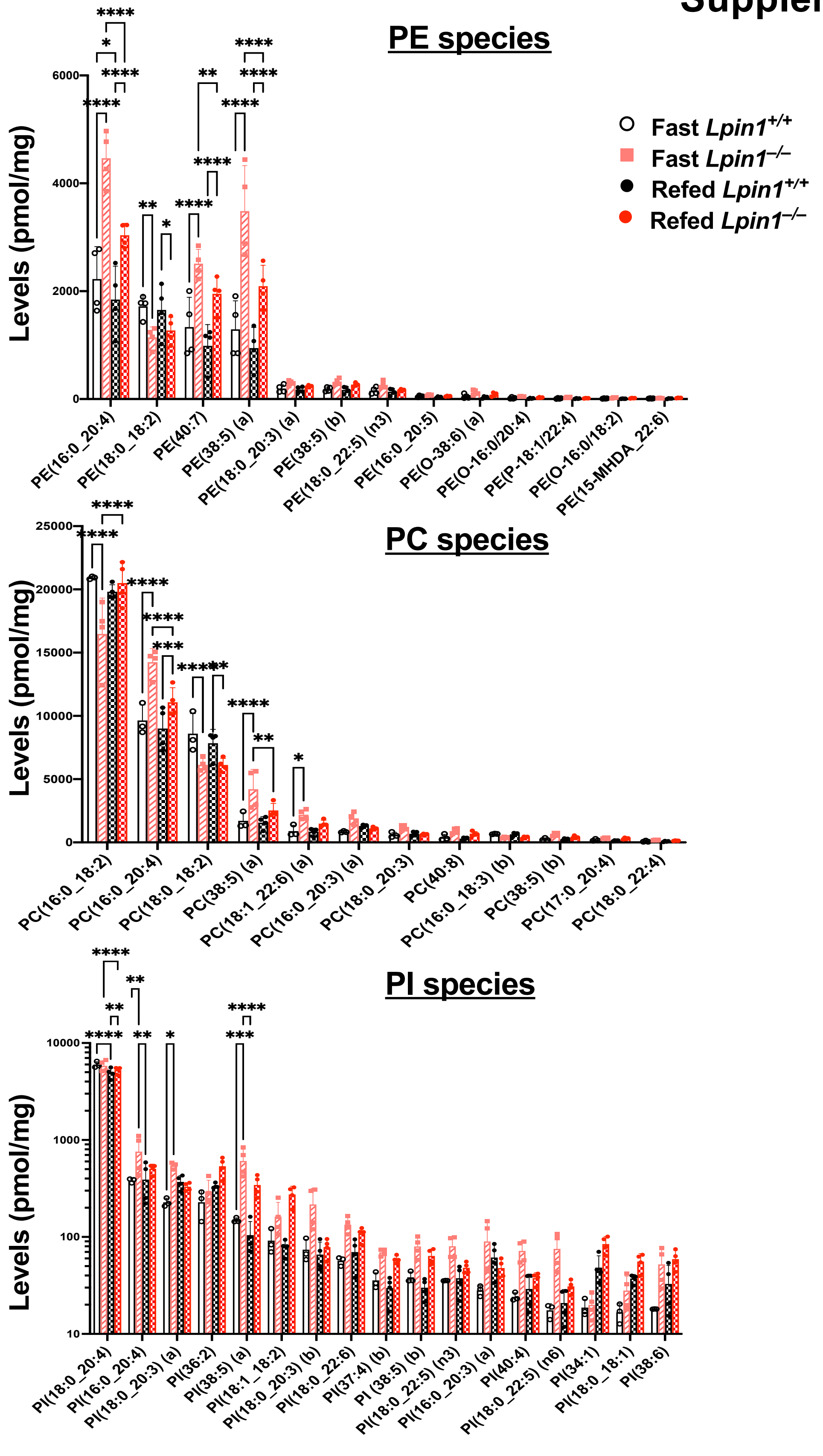

Supplemental Figure 6: PE, PC and PI species in fasted and refed liver. Phospholipid levels (pmol/mg) were determined by mass spectrometry for *Lpin1*$^{+/+}$ and *Lpin1*$^{-/-}$ mice under fasted and refed conditions. Asterisks indicate significant differences analyzed by 2-way ANOVA followed by t-test (n=4); *, p<0.05, **, p<0.01, ***, p<0.0001).

Full unedited gel for Figure 2B

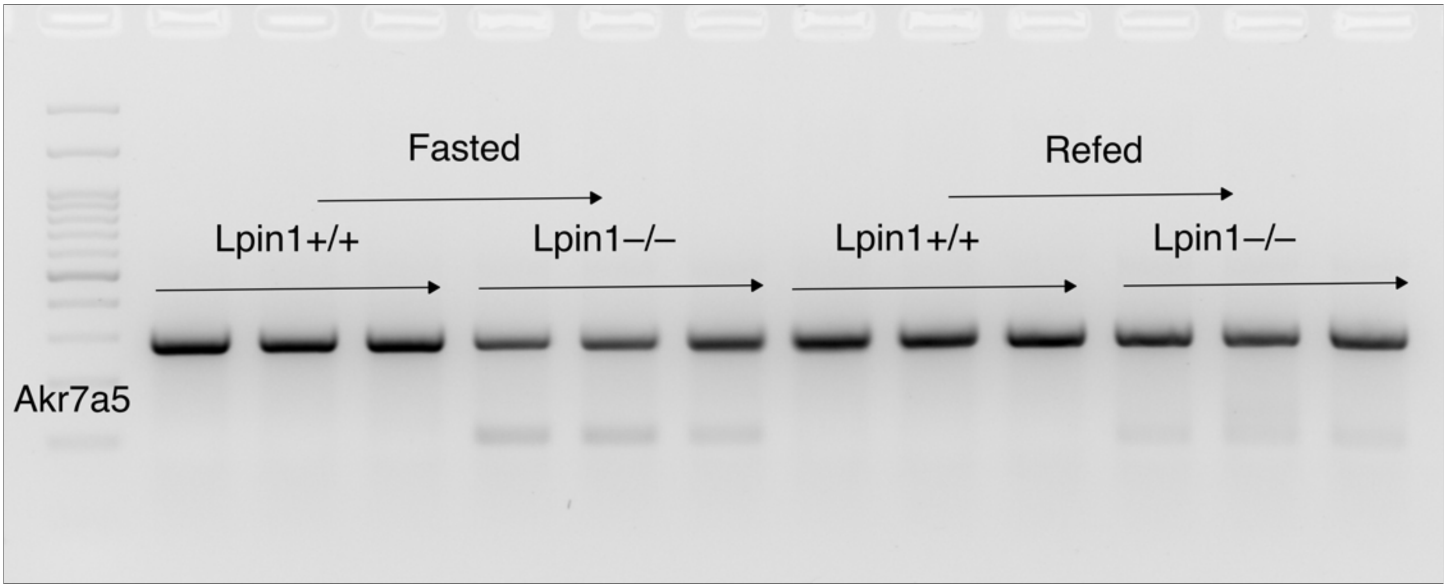

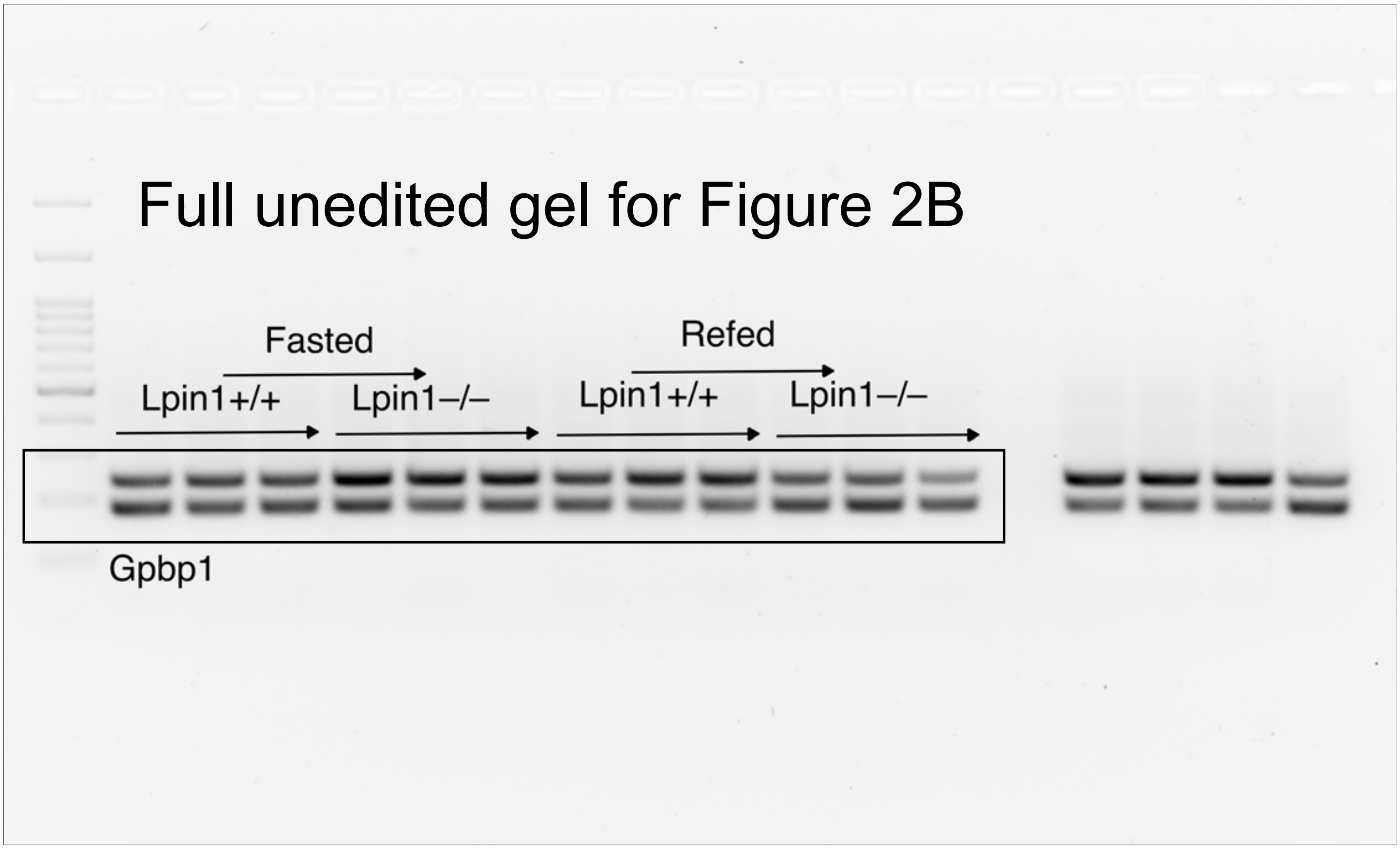
Full unedited gel for Figure 2B
Fasted
Refed
Lpin1+/+
Lpin1–/–
Lpin1+/+
Lpin1–/–
Gpbp1

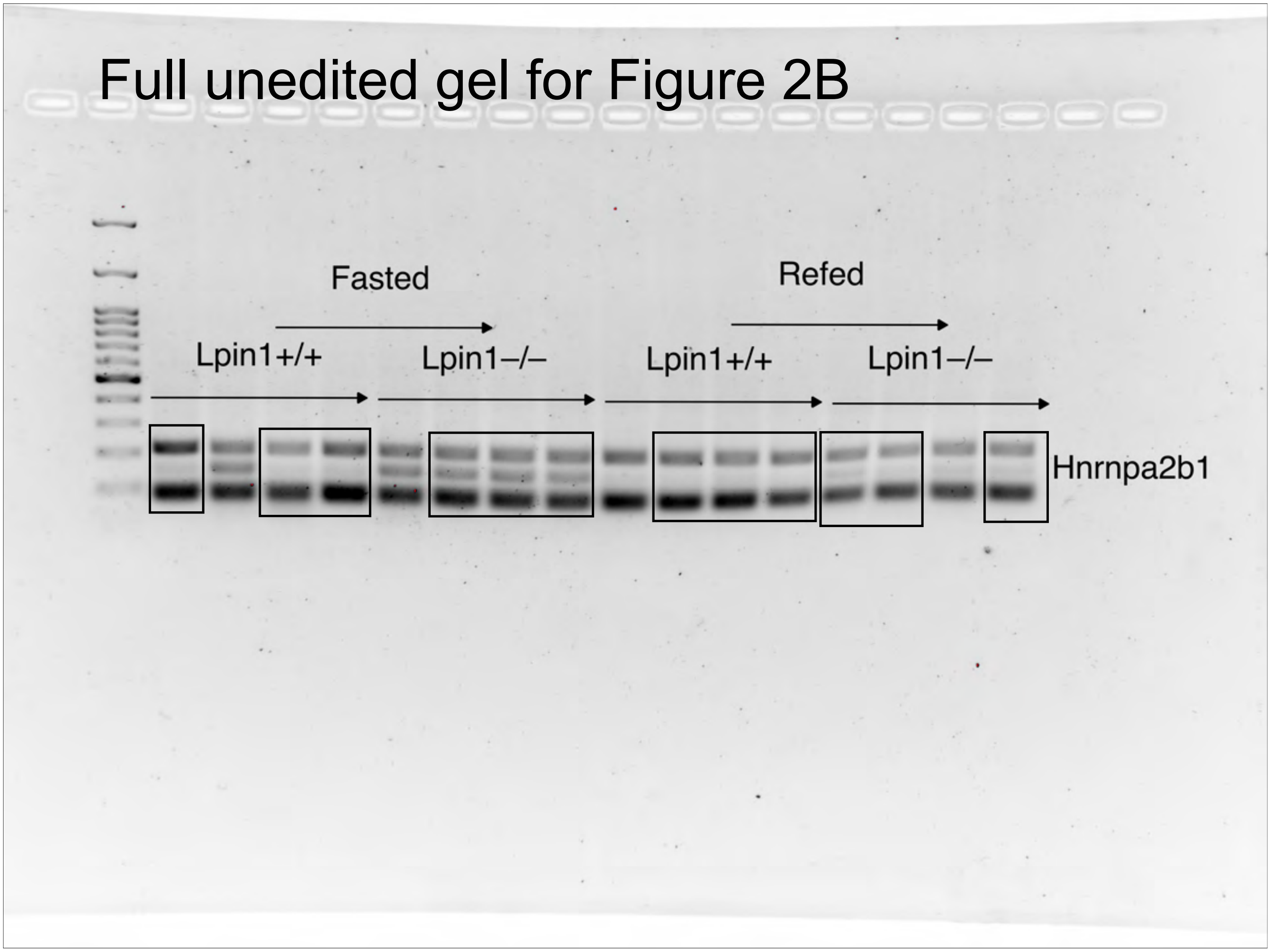
Full unedited gel for Figure 2B
Fasted
Refed
Lpin1+/+
Lpin1−/−
Lpin1+/+
Lpin1−/−
Hnrnpa2b1

Full unedited gel for Figure 2B

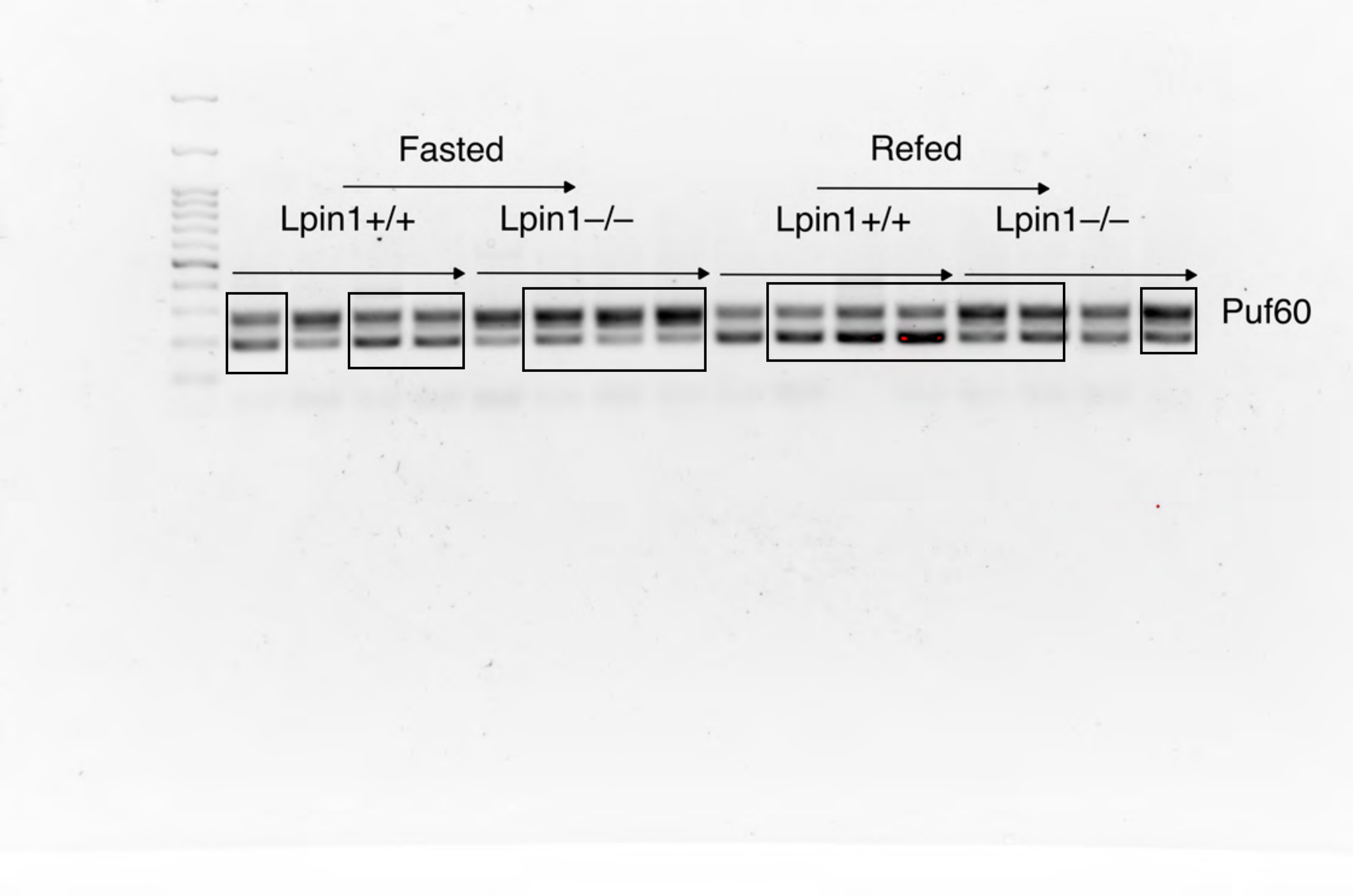

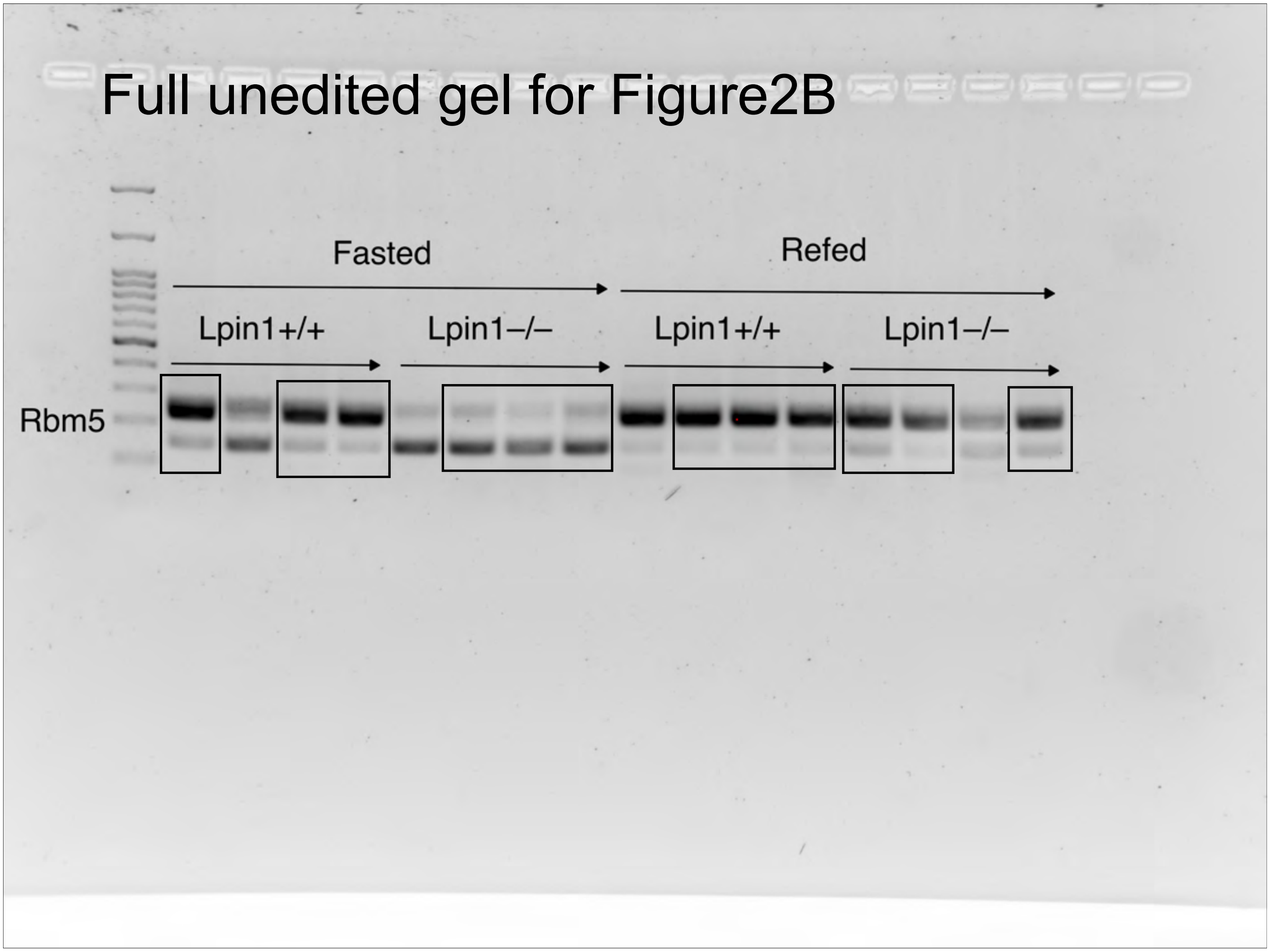
Full unedited gel for Figure2B
Fasted
Refed
Lpin1+/+
Lpin1–/–
Lpin1+/+
Lpin1–/–
Rbm5

Full unedited gel for Figure 2B

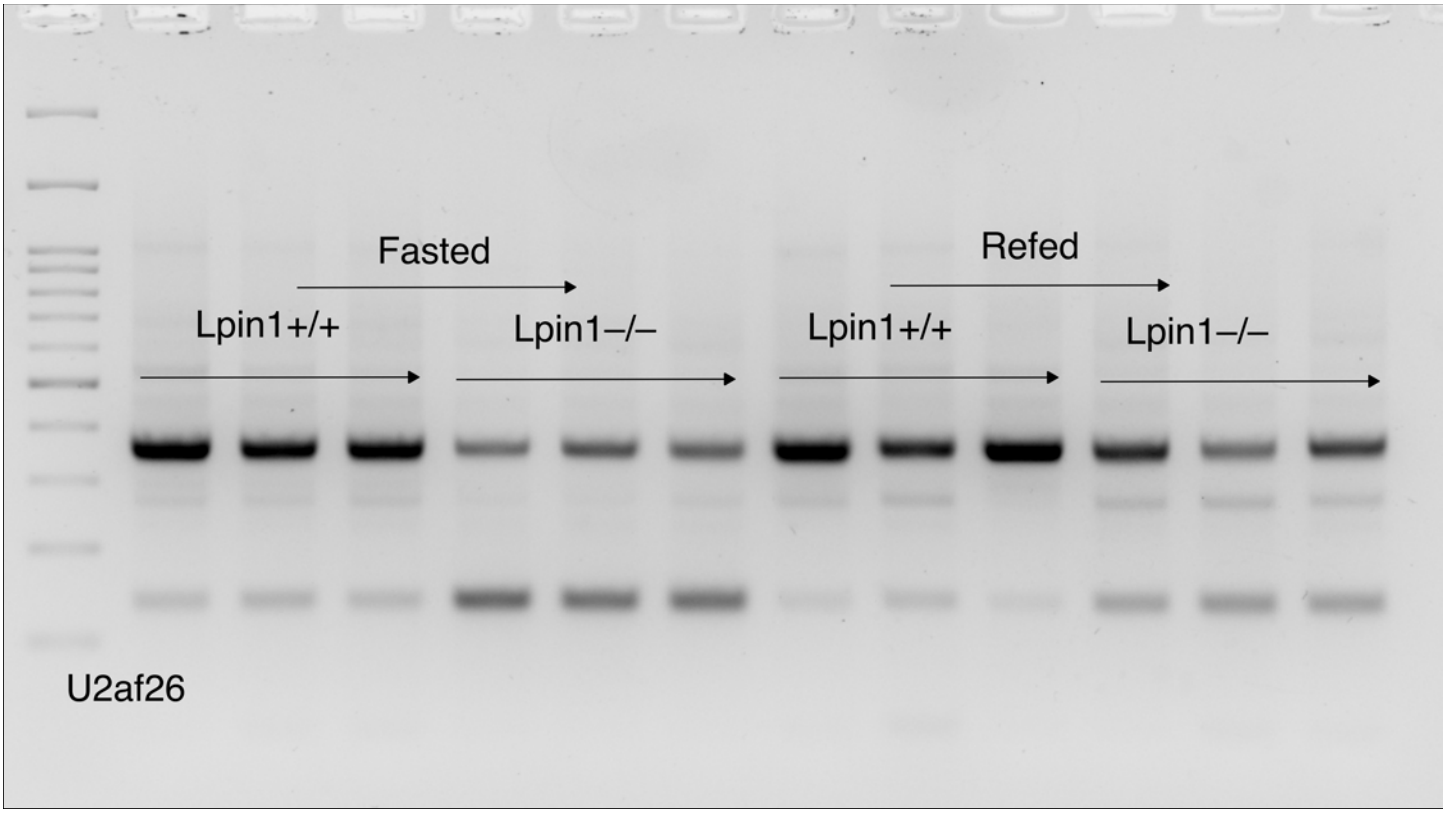

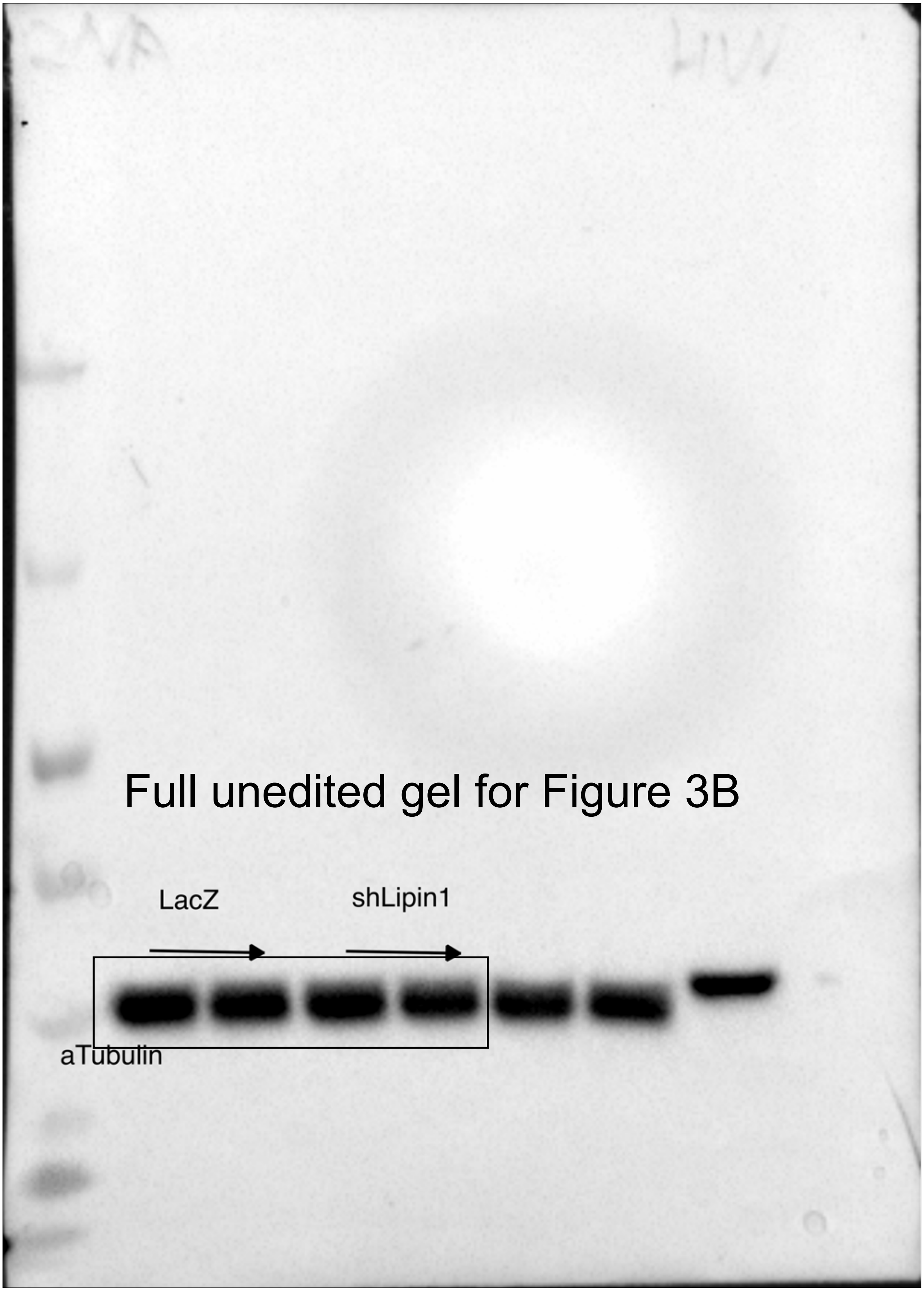
Full unedited gel for Figure 3B
LacZ
shLipin1
aTubulin

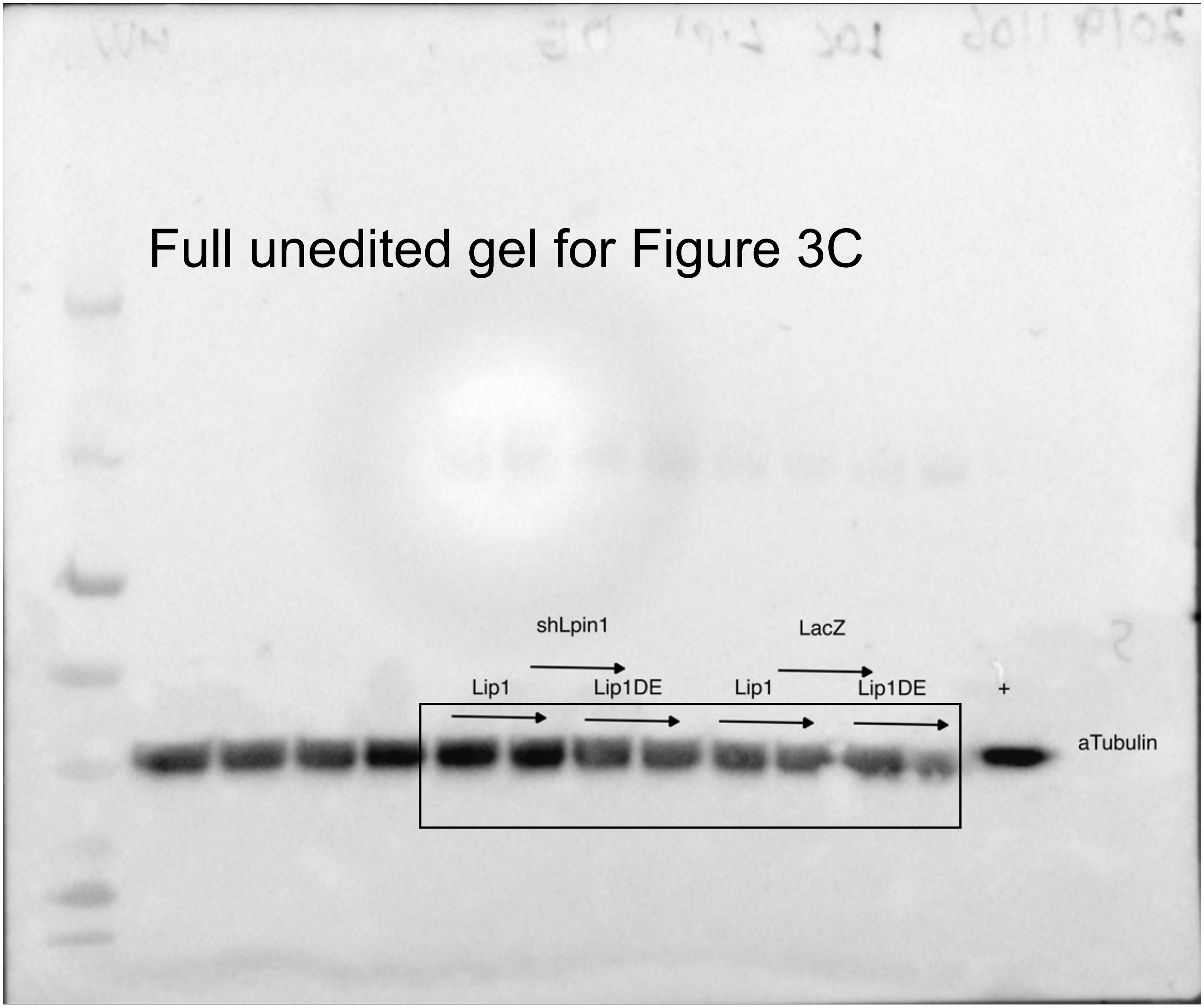
Full unedited gel for Figure 3C
shLpin1
LacZ
Lip1
Lip1DE
Lip1
Lip1DE
+
aTubulin

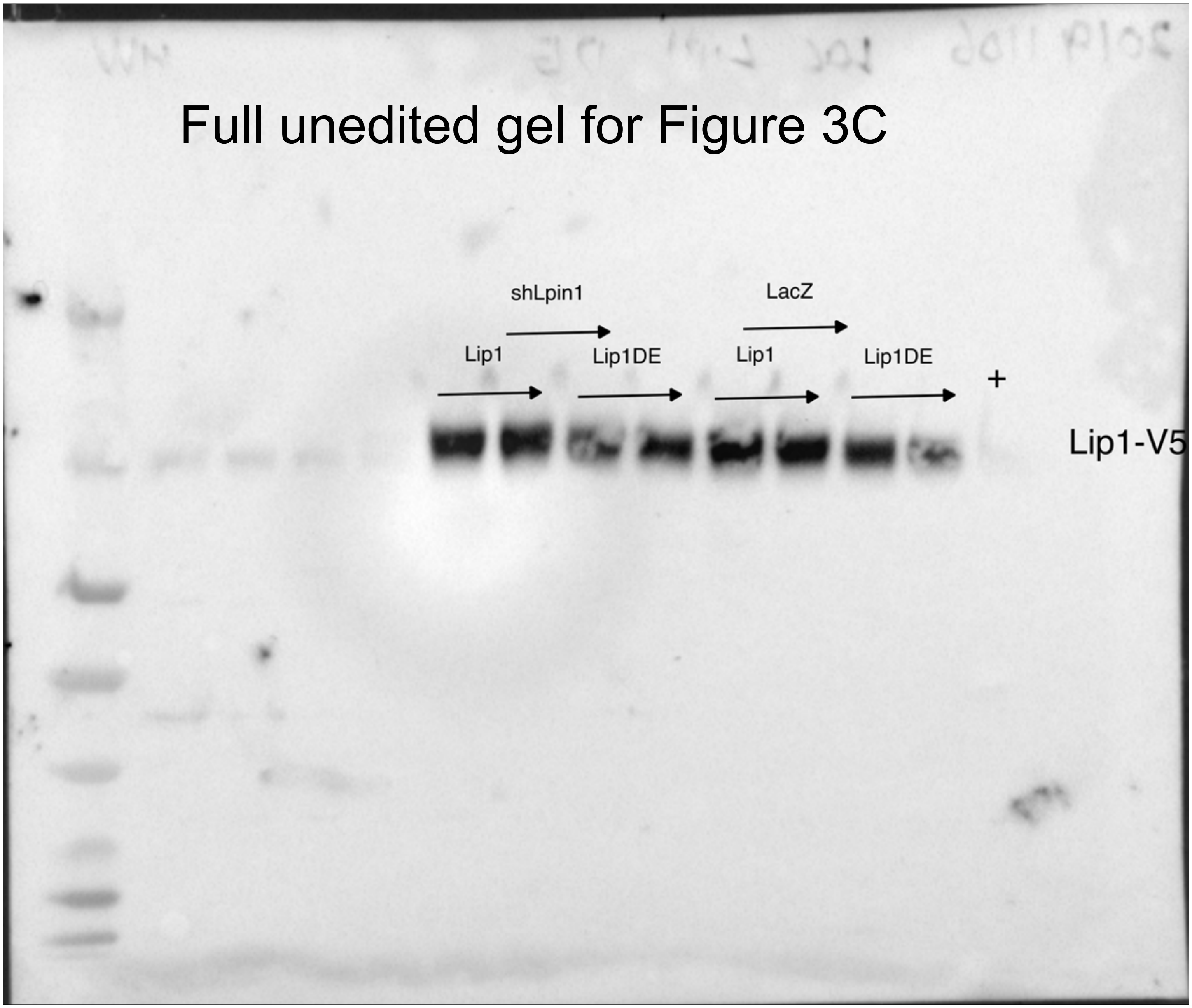
Full unedited gel for Figure 3C
shLpin1
LacZ
Lip1
Lip1DE
Lip1
Lip1DE
+
Lip1-V5

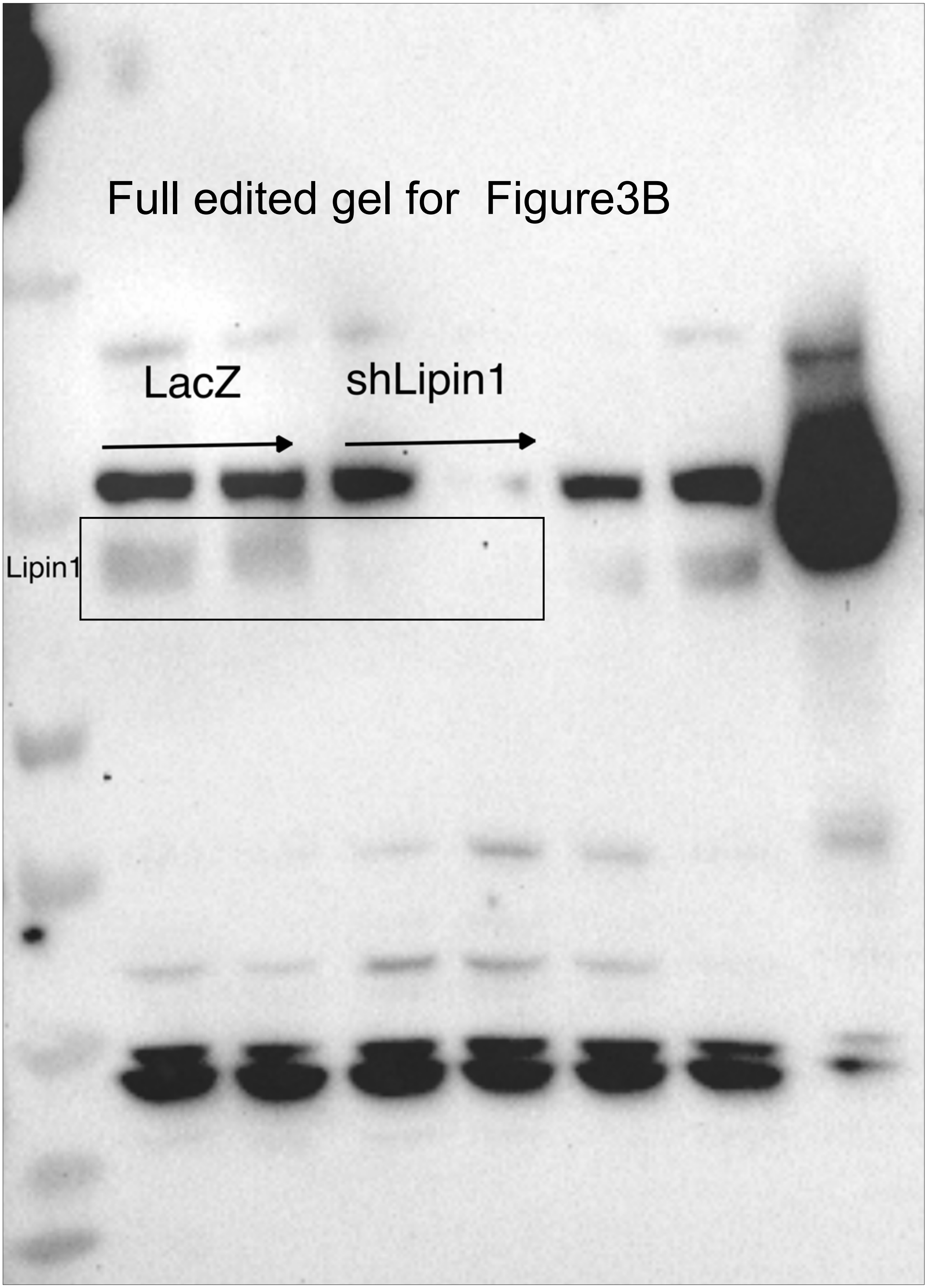

Location: /Volumes/HUAN W/20191025 hPLD1 in HepG2 lipin1 in ShRNALipin1_2 hepa16/2nd take
Printed: 4/2/21 1:42 PM

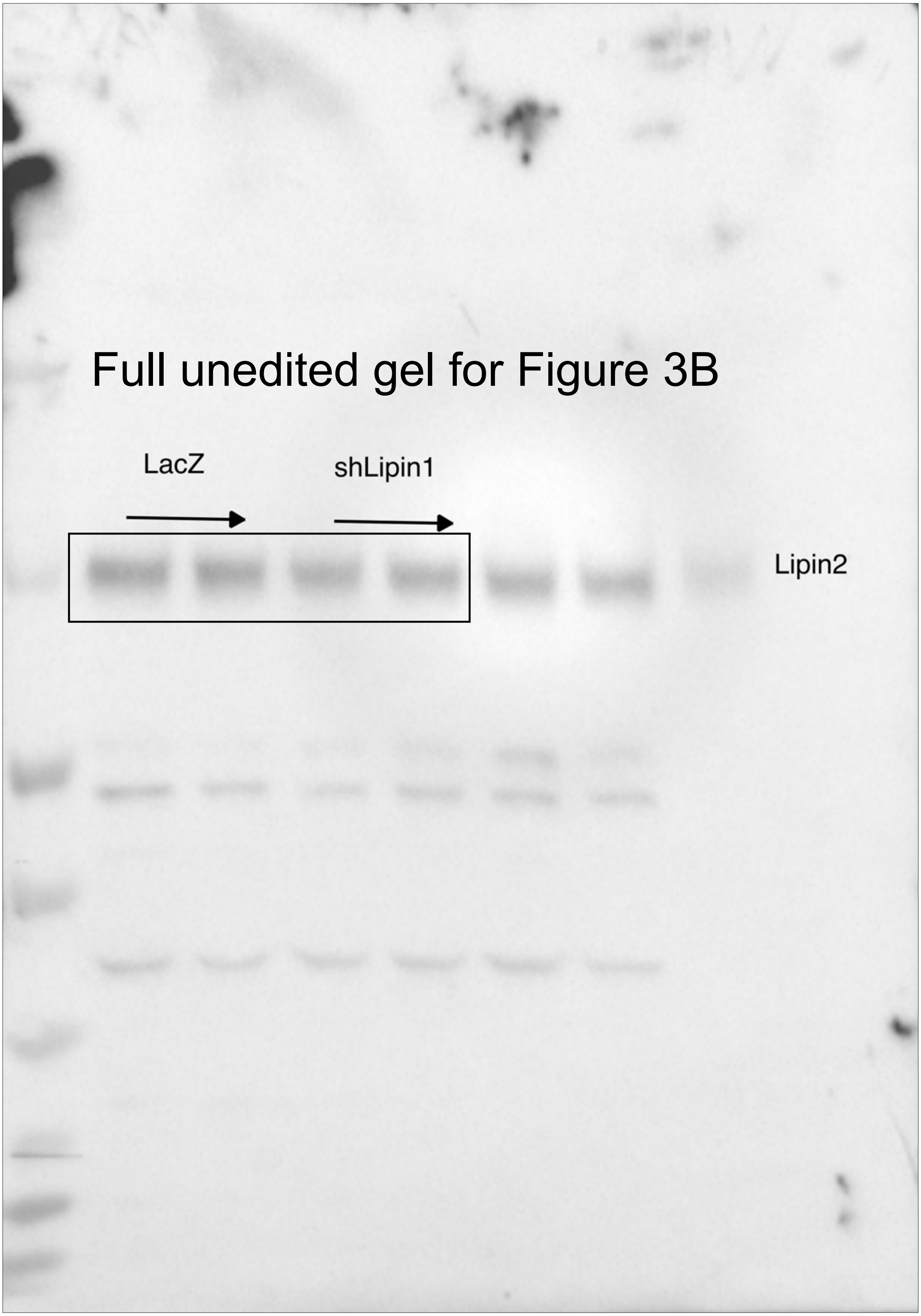
Full unedited gel for Figure 3B
LacZ
shLipin1
Lipin2

Full unedited gel for Figure 3D

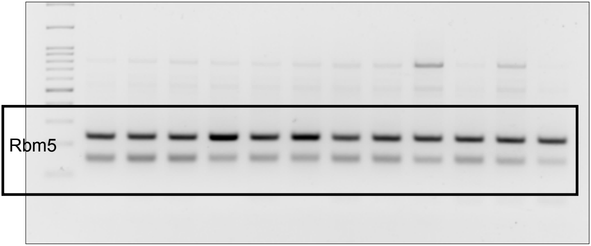

Full unedited gel for Figure 3D

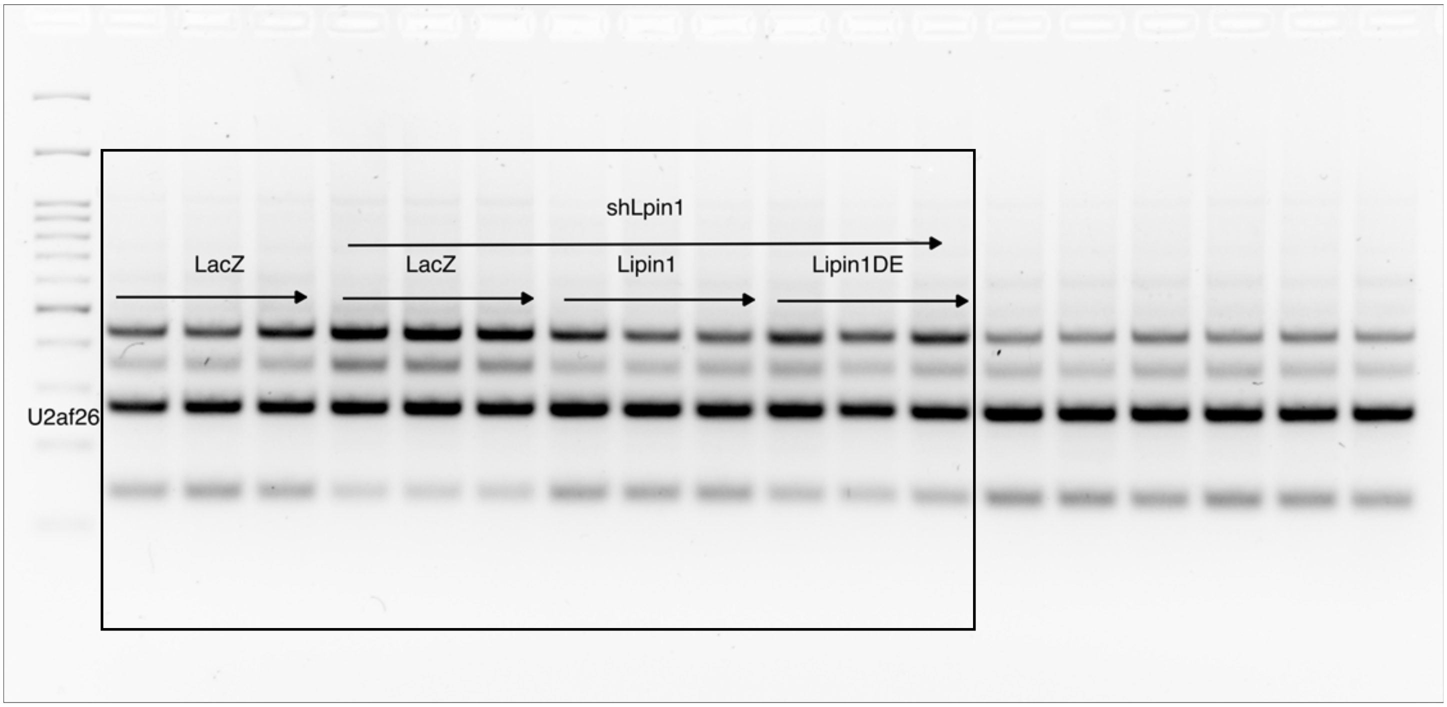

Full unedited gel for Figure 4C

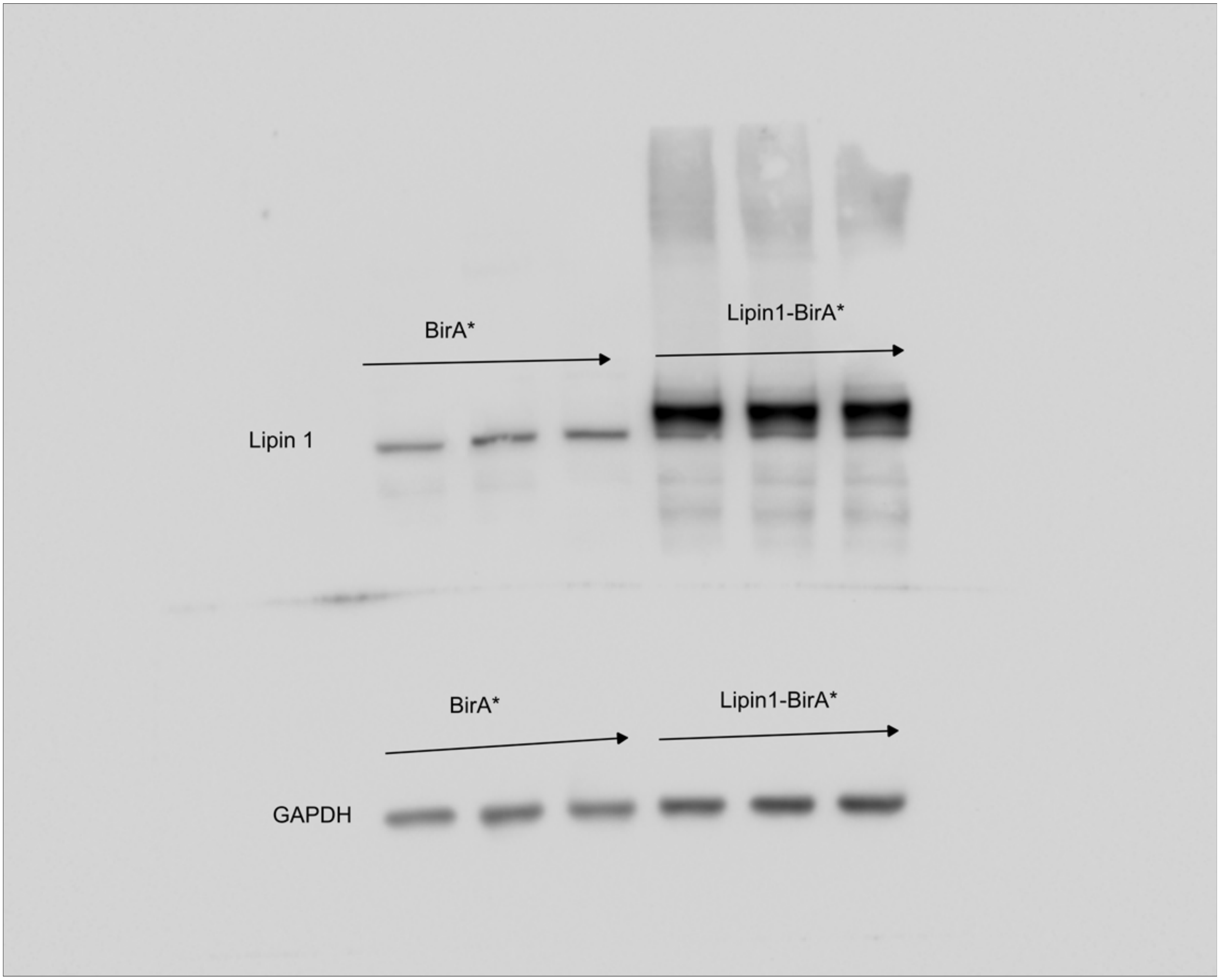

Full unedited gel for Figure 4C

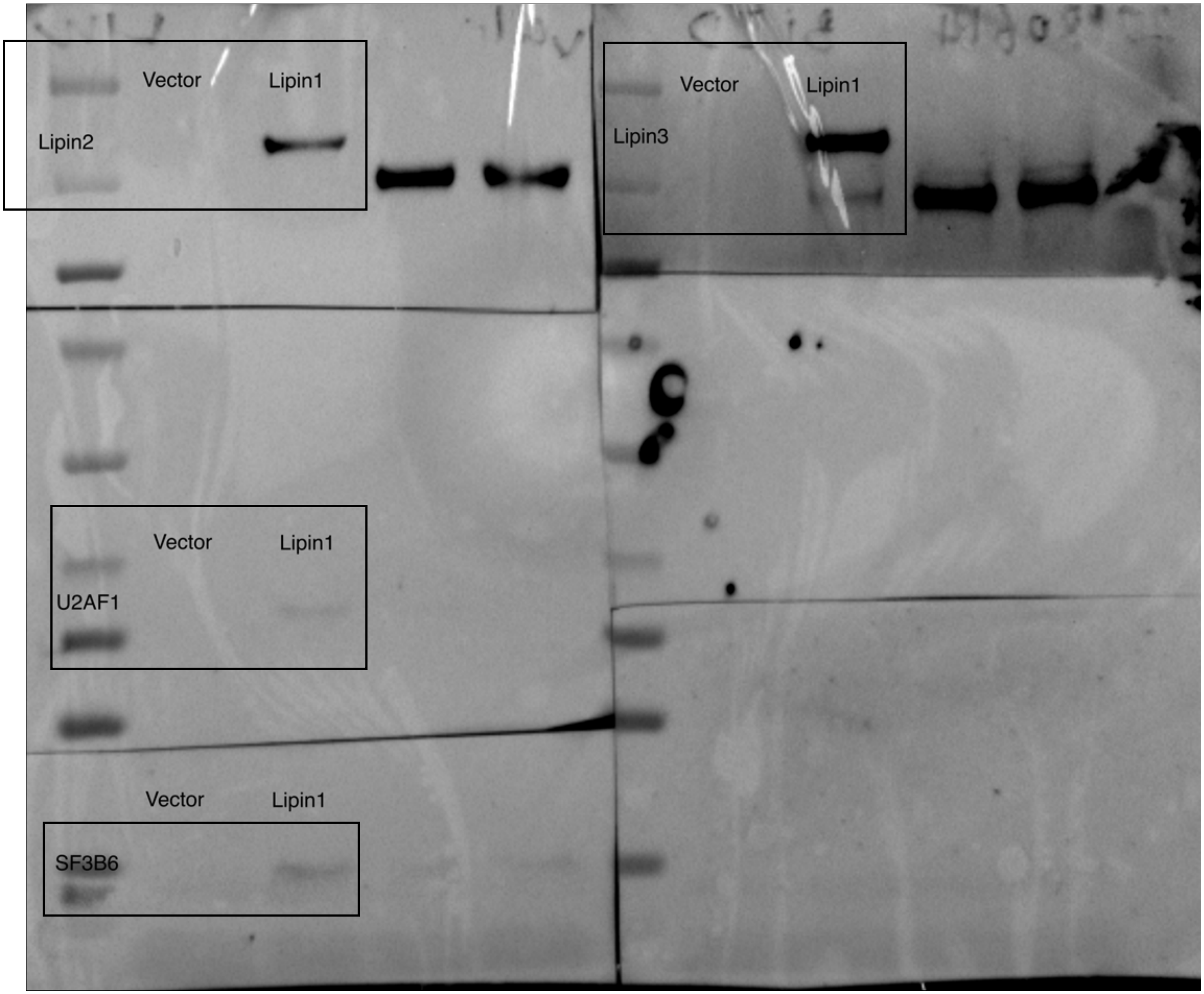

Full unedited gel for Figure 4D

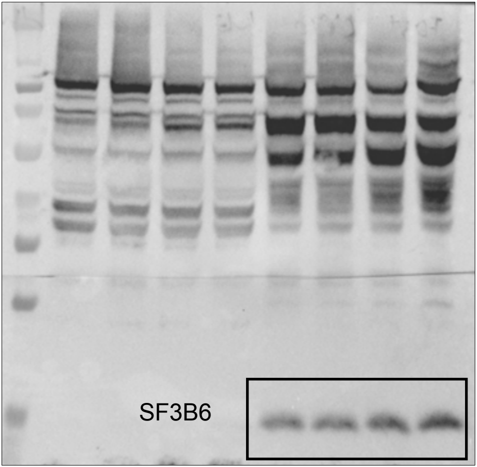

Full unedited gel for Figure 4C

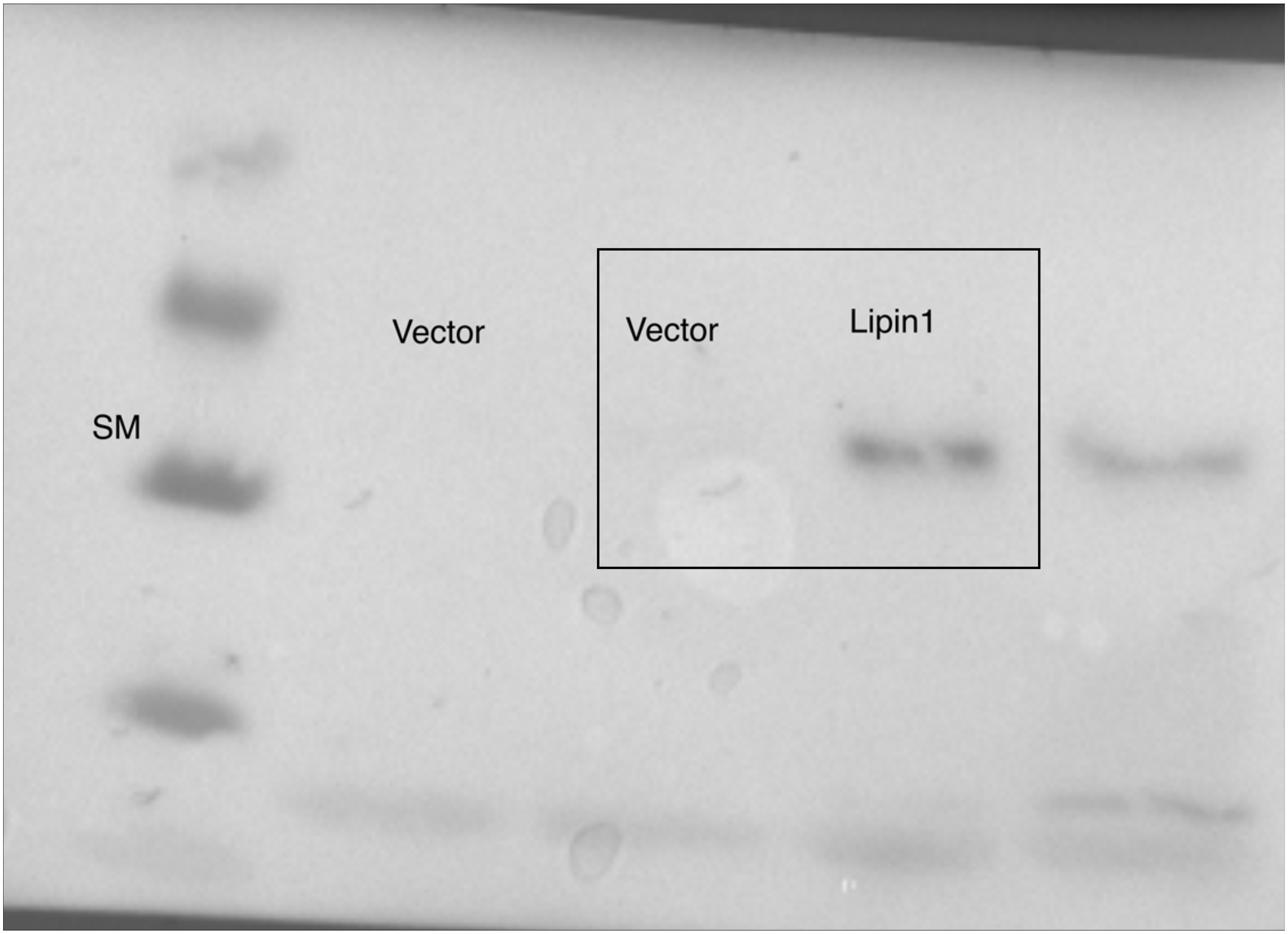

Full unedited gel for Figure 4D

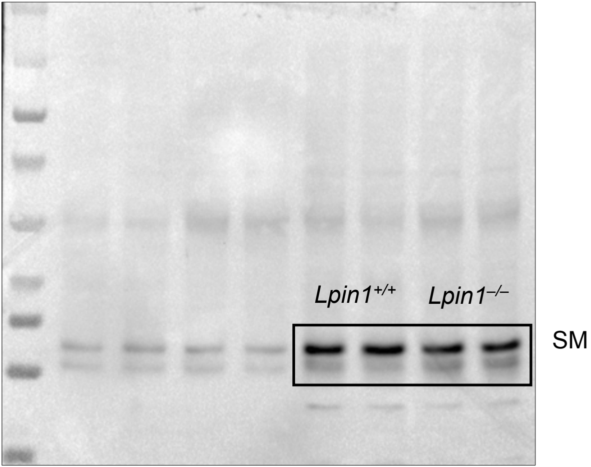

Full unedited figure for Figure 4C

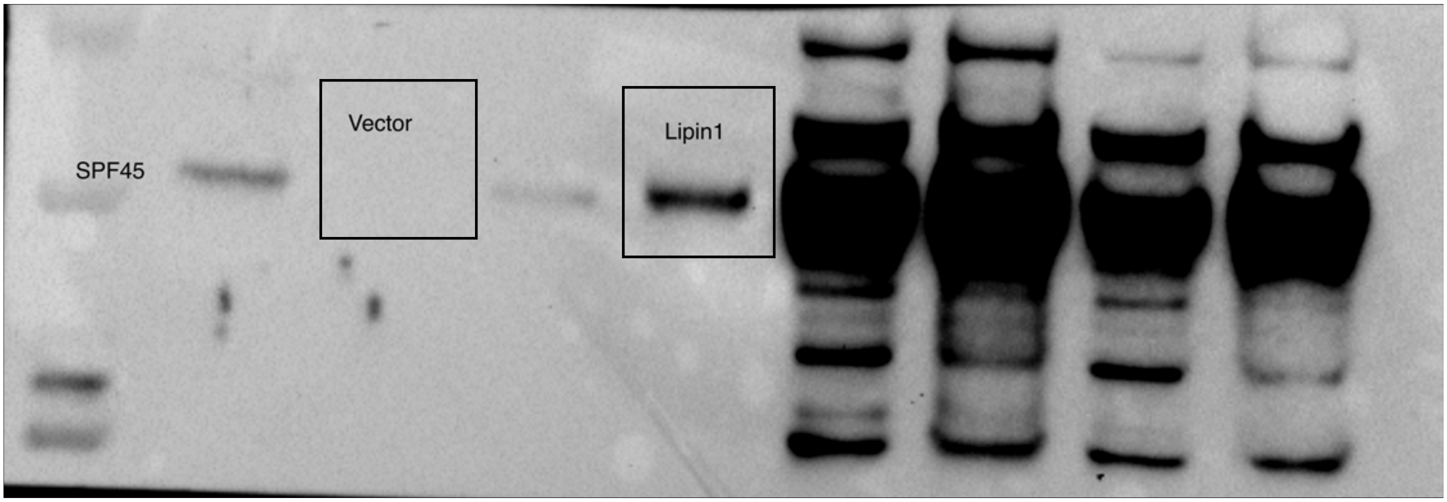

Full unedited gel for Figure 4D

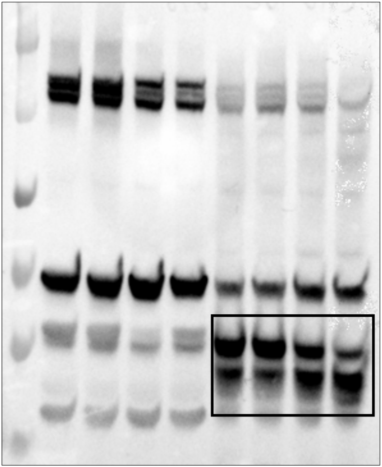

Full unedited gel for Figure 4D

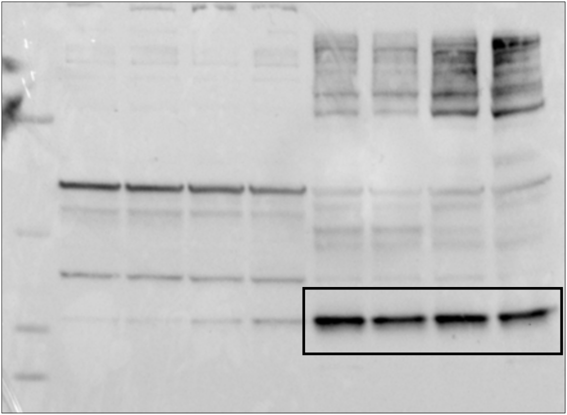

Full unedited gel for Figure 4D

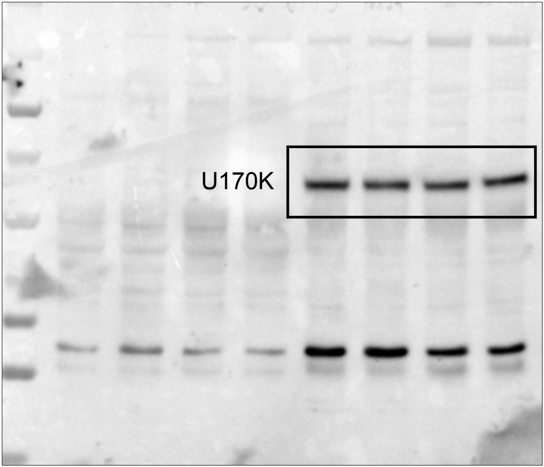

Full unedited gel for Figure 5F

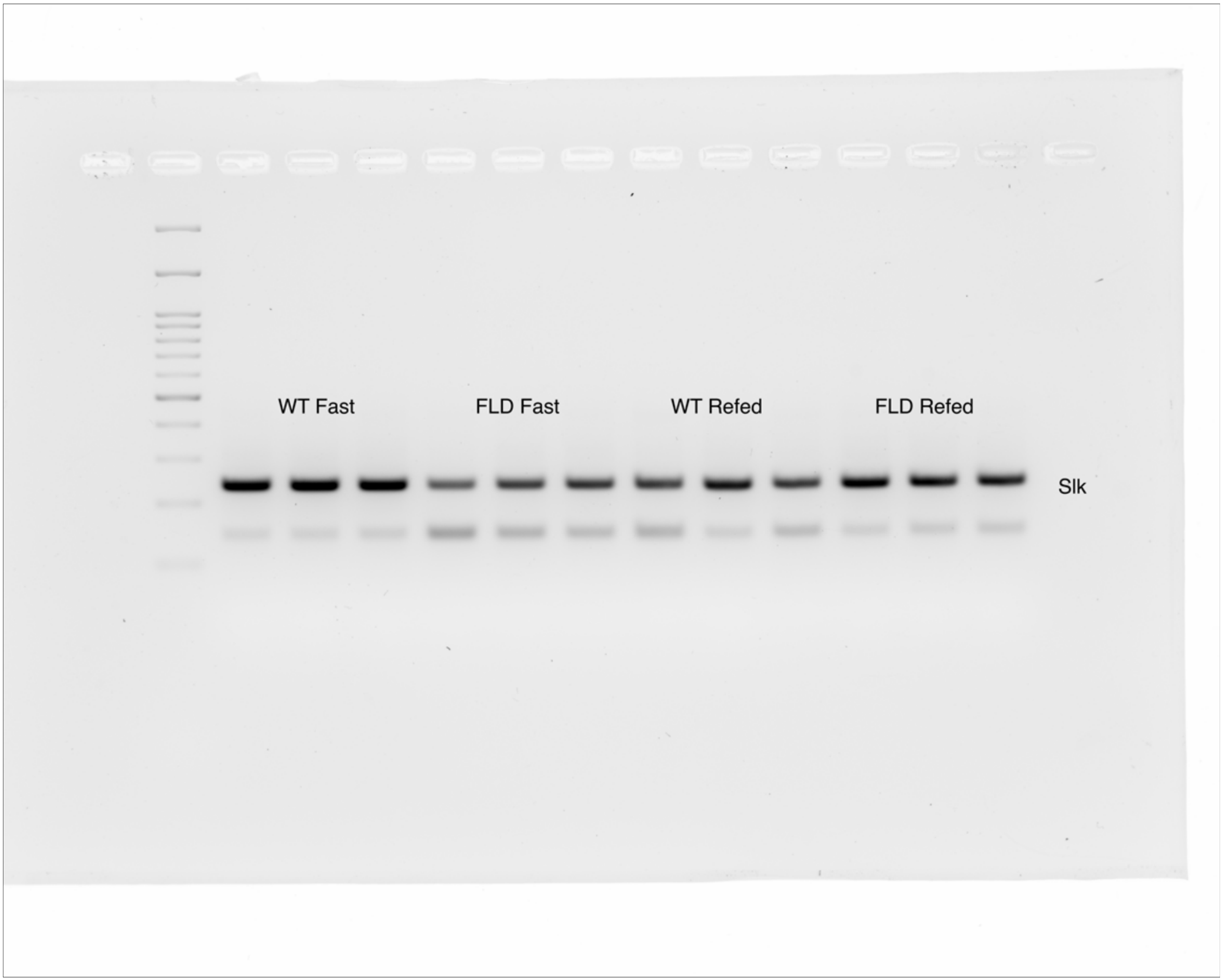

Full unedited gel for Figure 5F

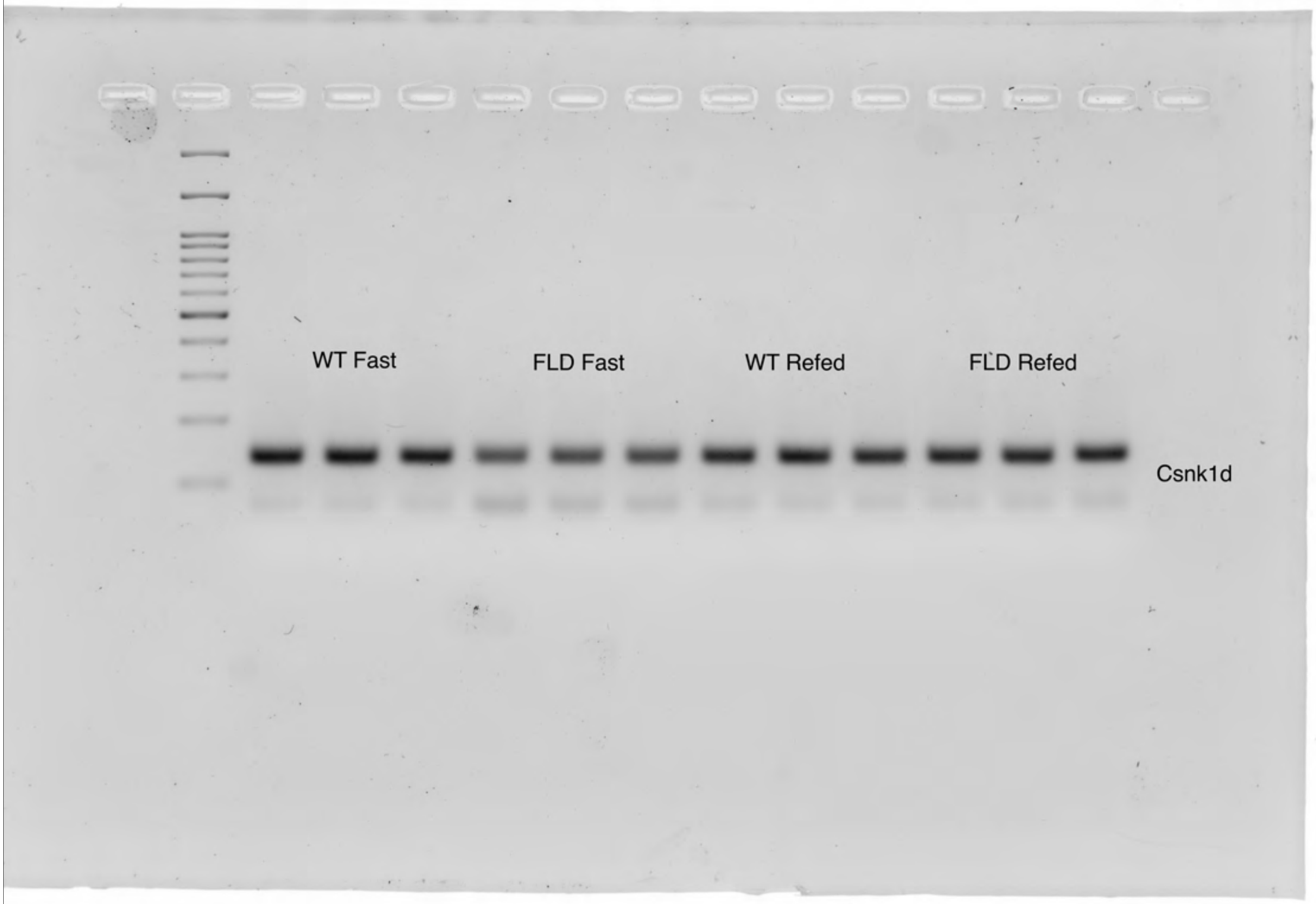

Full unedited gel for Figure 7E

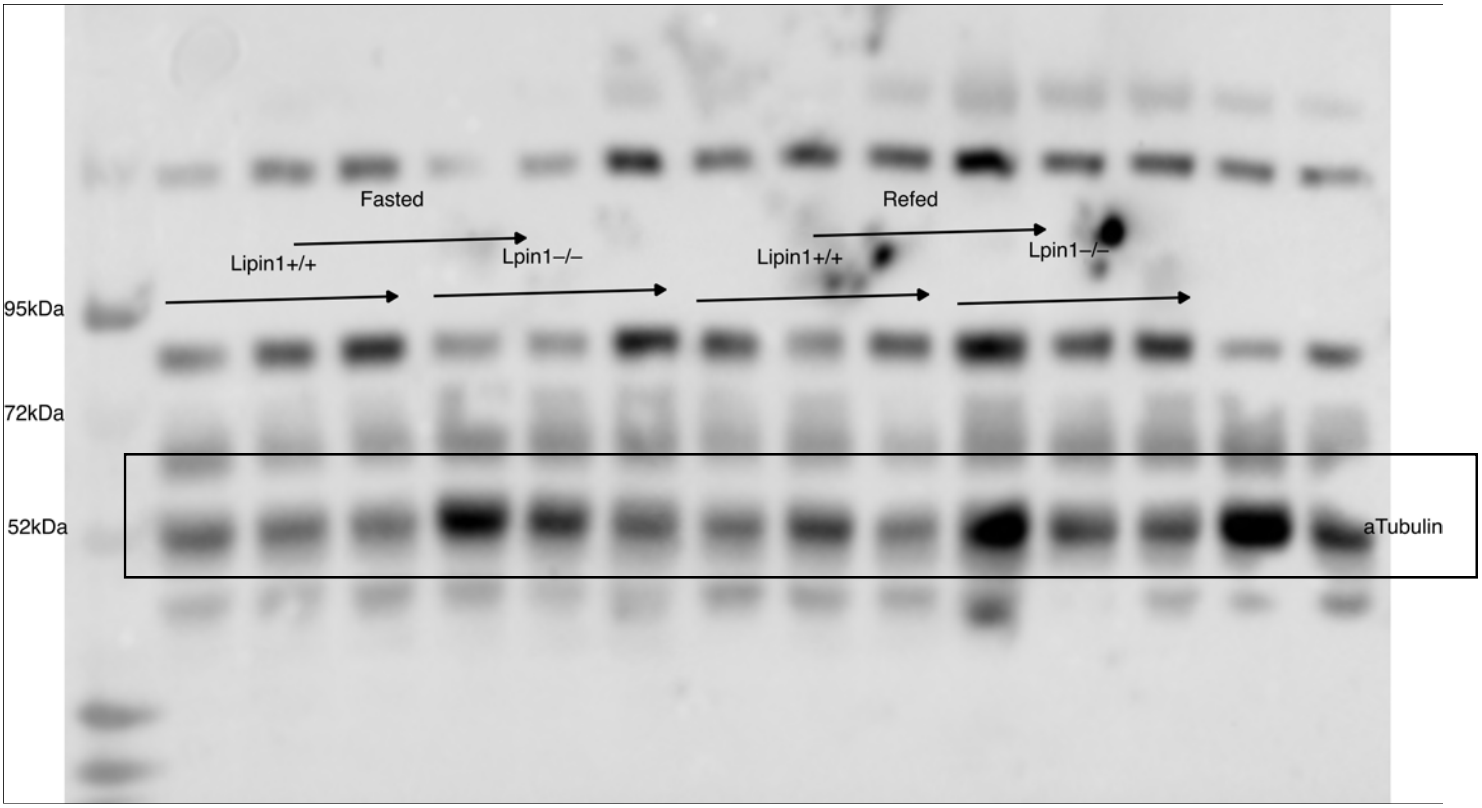

Location: /Volumes/LAB_HW/ChemiDoc Images 2021-05-27_17.42.20/2021-05-27 13h41m59s Huan
Printed: 5/28/21 12:14 PM

Full unedited gel for Figure 7C

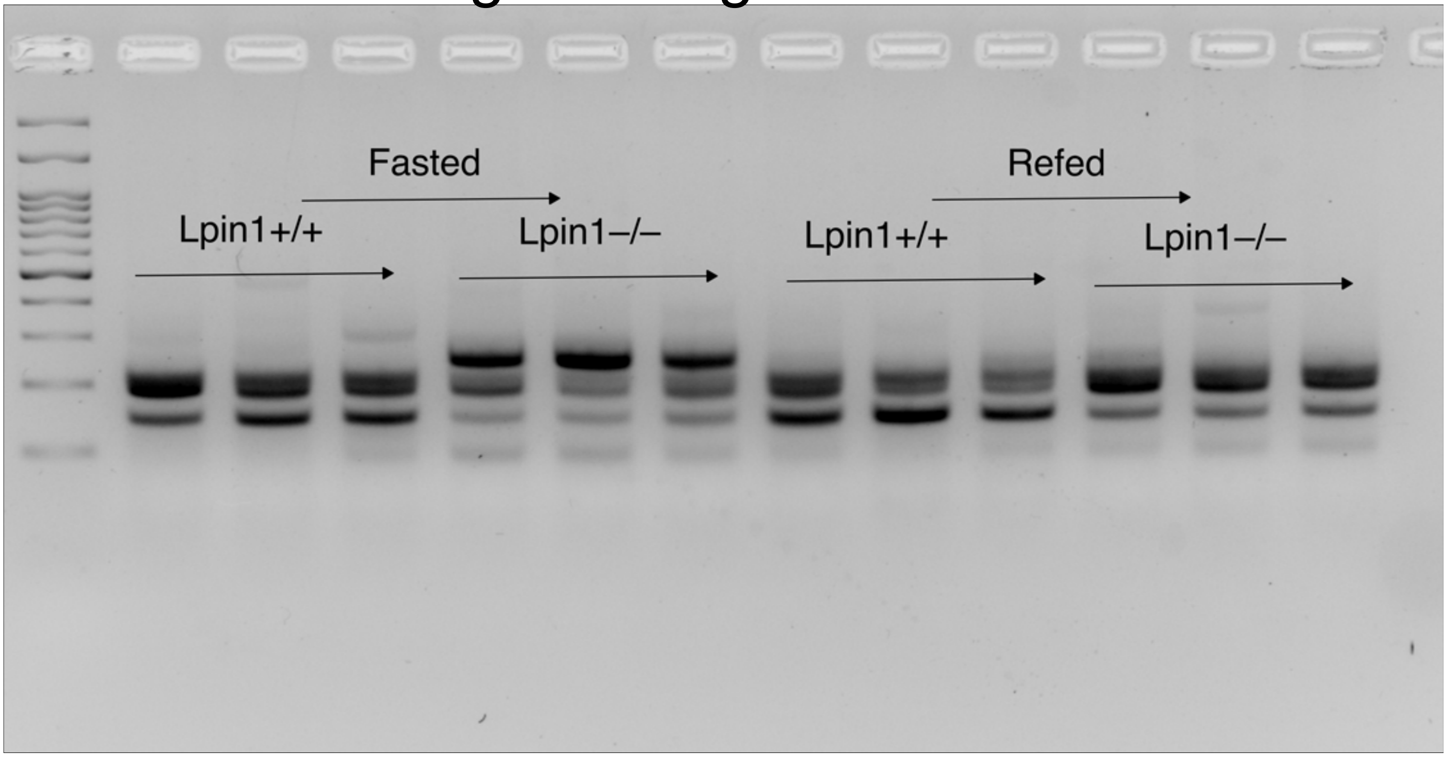

Full unedited gel for Figure 7E

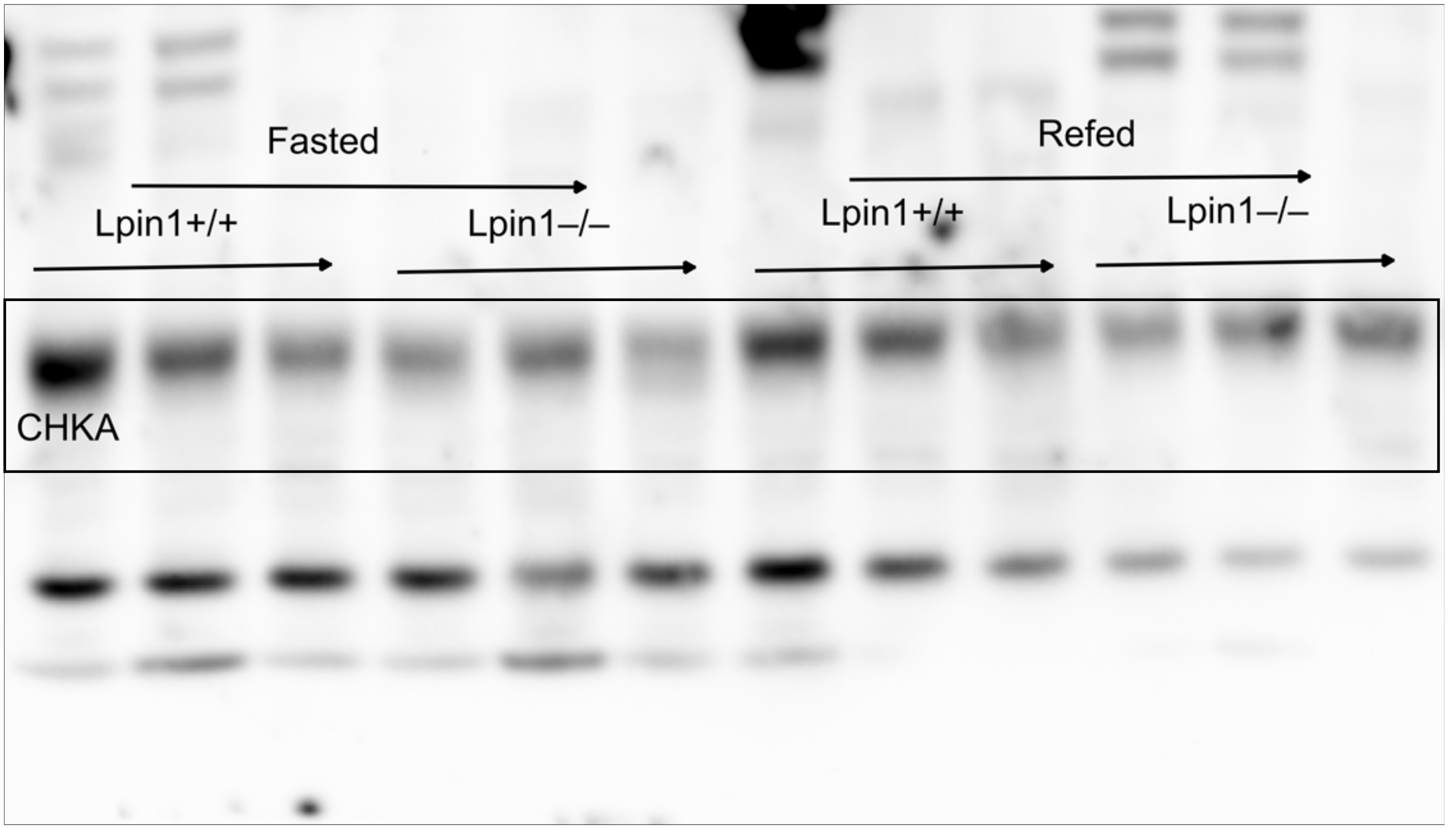

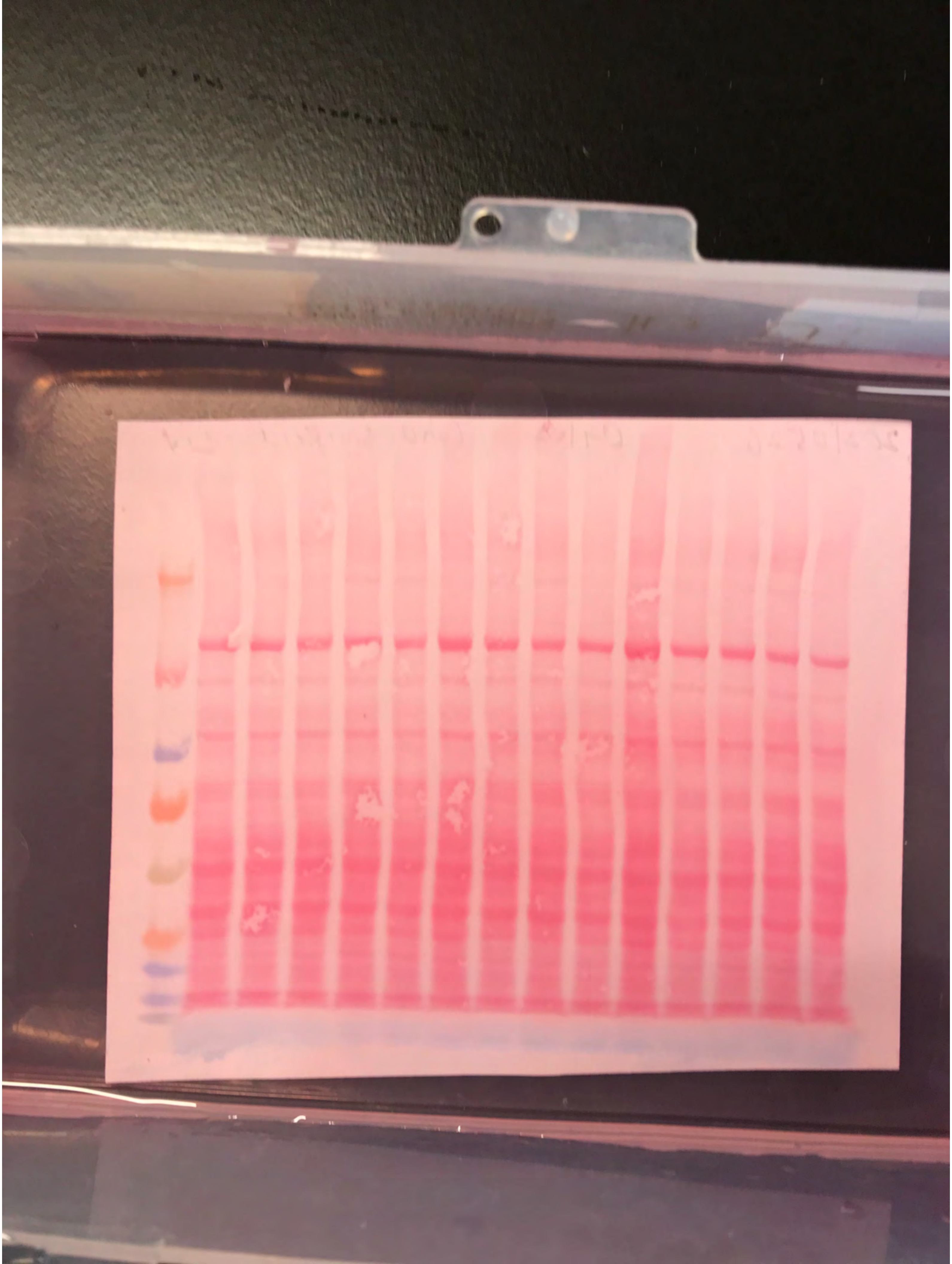

Full unedited gel for Supplemental Figure 3C

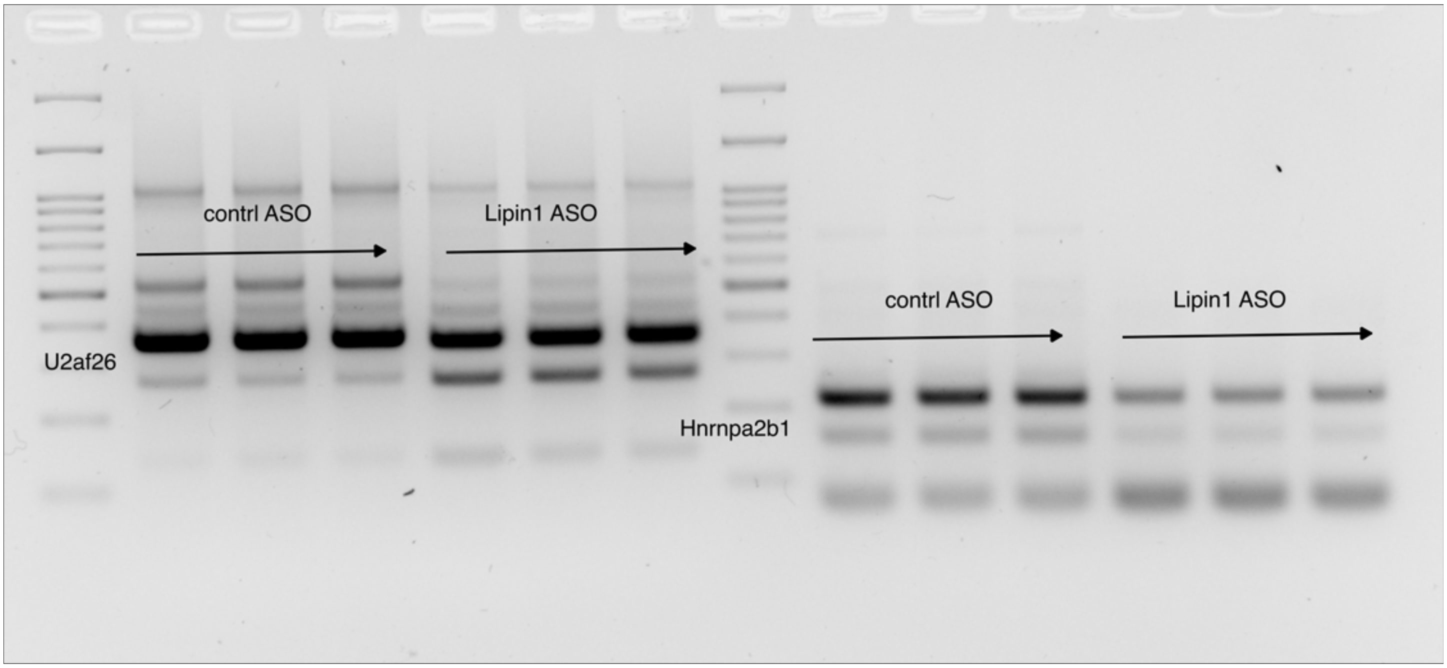

Full unedited gel for Supplemental Figure 3B

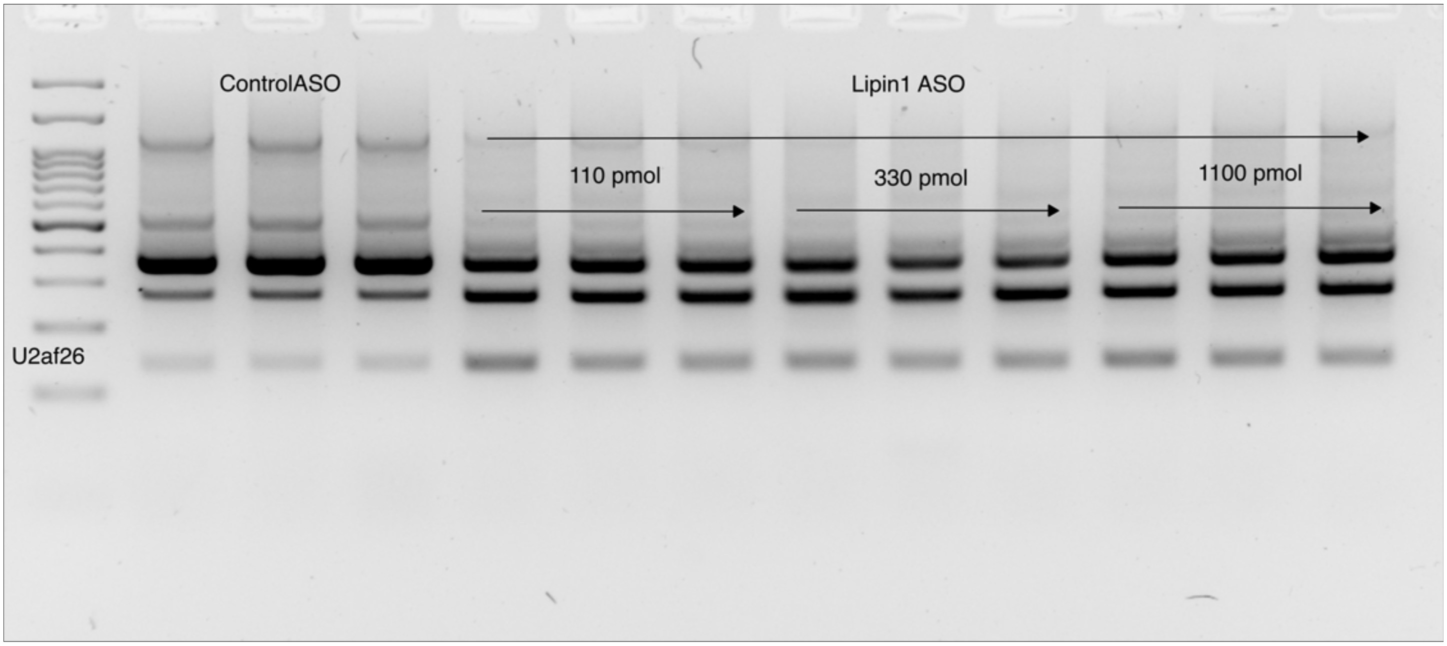

Full unedited gel for Supplemental Figure 4A

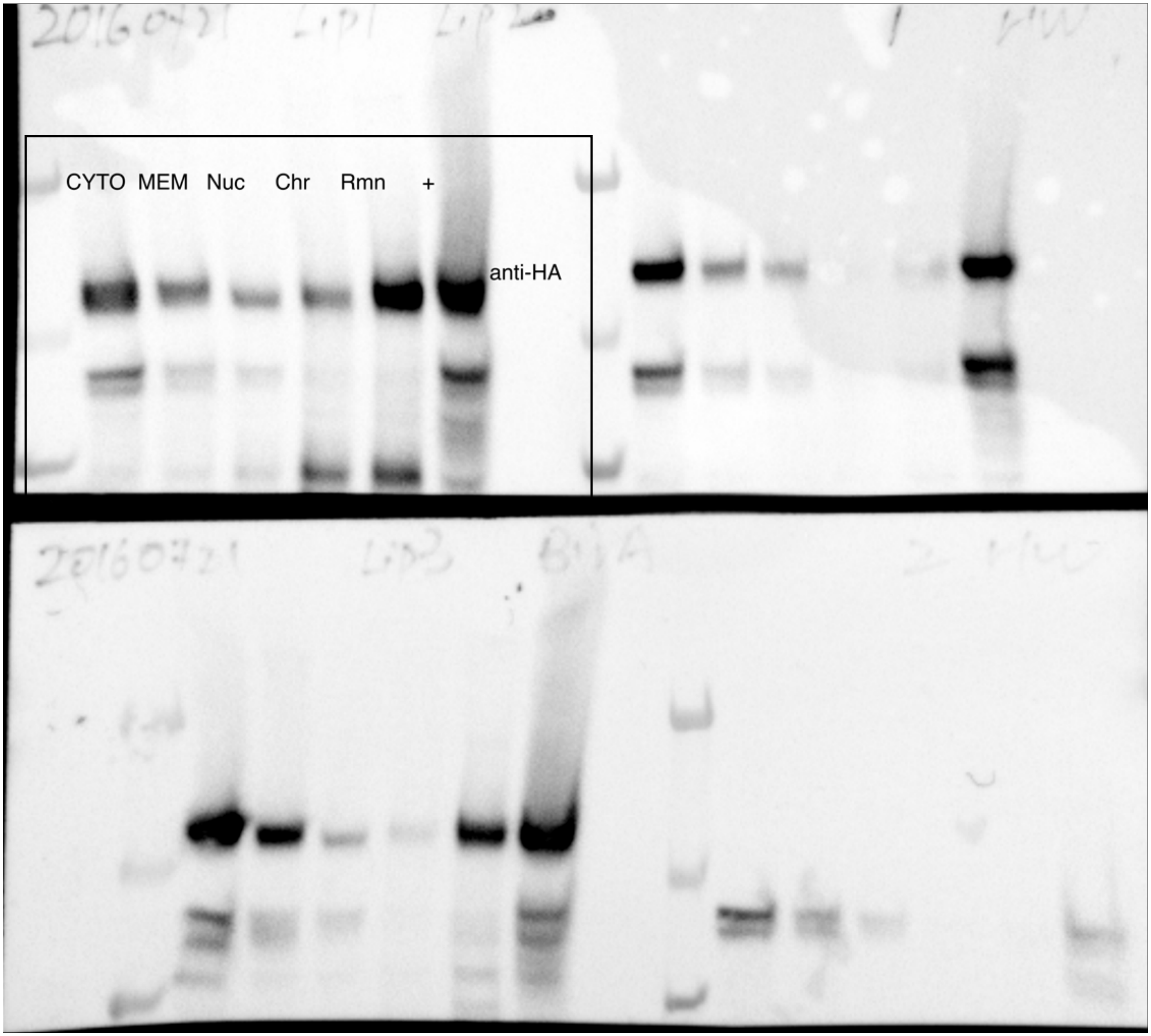

Full unedited gel for Supplemental Figure 4A

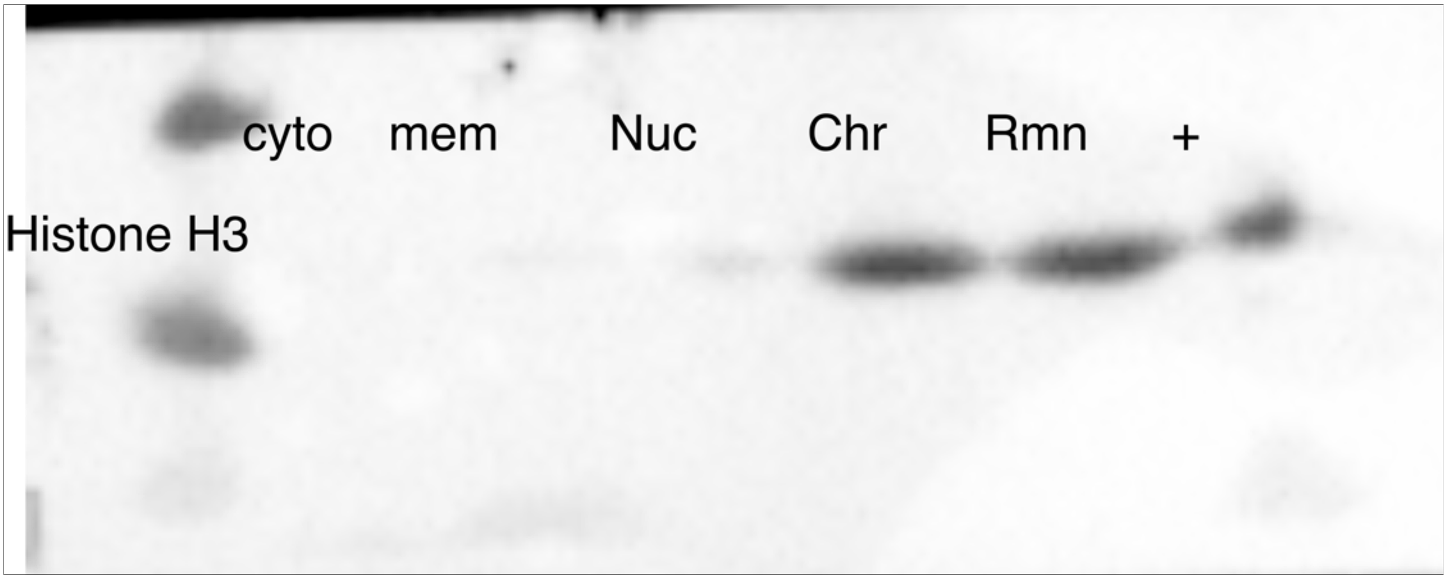

Full unedited gel for Supplemental Figure 4A

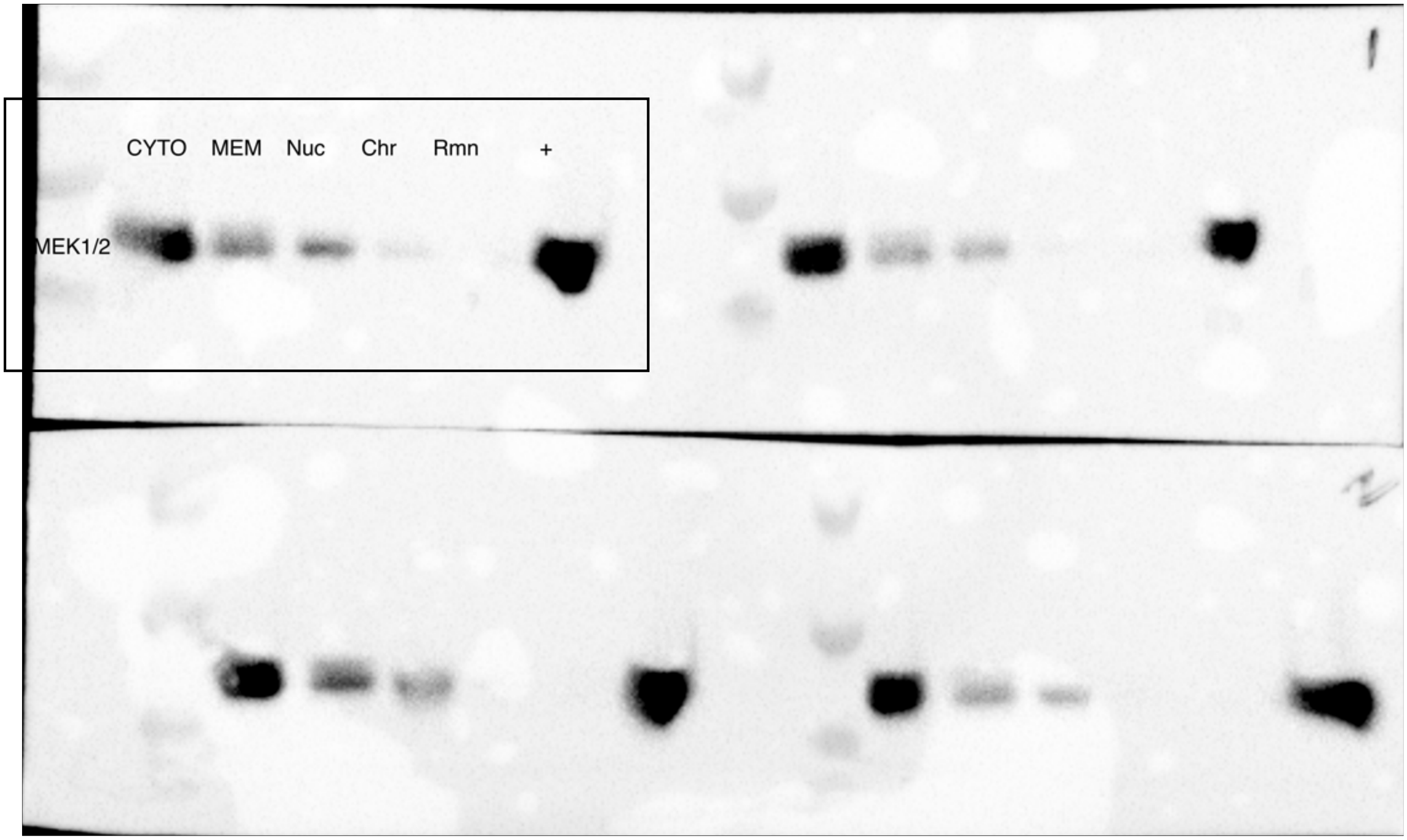

Full unedited gel for Supplemental Figure 4D

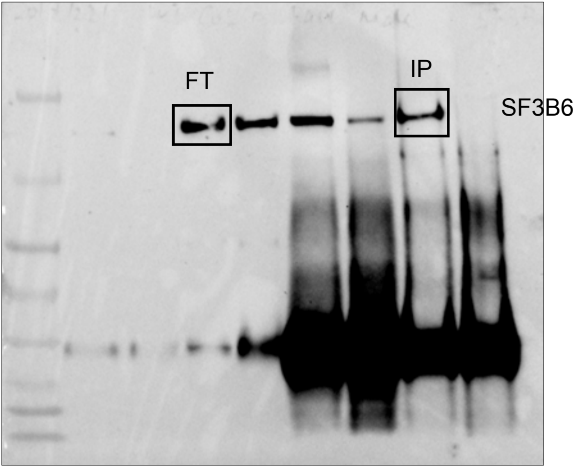

Full unedited gel for Supplemental Figure 4D

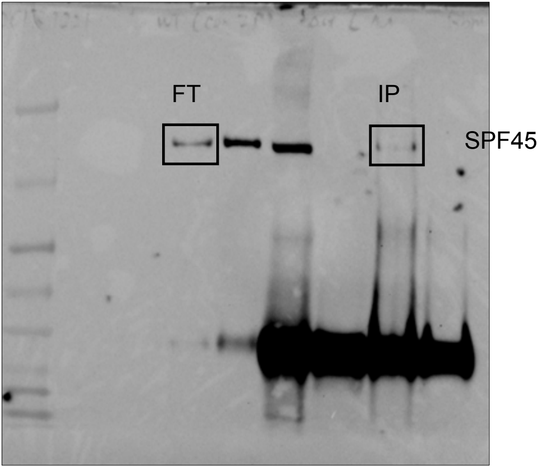

Supplement: Supplemental data [file jciinsight-6-150114-s251.pdf]
